# Obesity Resistance and Enhanced Insulin Sensitivity in *Ahnak* ^-/-^ Mice Fed a High Fat Diet Are Related to Impaired Adipogenesis and Increased Energy Expenditure

**DOI:** 10.1371/journal.pone.0139720

**Published:** 2015-10-14

**Authors:** Jae Hoon Shin, Il Yong Kim, Yo Na Kim, Sun Mee Shin, Kyung Jin Roh, Seo Hyun Lee, Mira Sohn, Soo Young Cho, Sang Hyuk Lee, Chang-Yong Ko, Han-Sung Kim, Cheol Soo Choi, Yun Soo Bae, Je Kyung Seong

**Affiliations:** 1 Laboratory of Developmental Biology and Genomics, College of Veterinary Medicine, and BK21 Program for Veterinary Science, Seoul National University, Seoul, South Korea; 2 Korea Mouse Phenotyping Center (KMPC), Seoul National University, Seoul, South Korea; 3 Division of Life Sciences, Ewha Womans University, Seoul, South Korea; 4 Lee Gil Ya Cancer and Diabetes Institute and Division of Endocrinology Gil Medical Center, Gachon University of Medicine and Science, Incheon, South Korea; 5 Ewha Research Center for Systems Biology, Ewha Womans University, Seoul, South Korea; 6 Department of Biomedical Engineering, College of Health Science, Institute of Medical Engineering, Yonsei University, Wonju, South Korea; 7 Interdisciplinary Program for Bioinformatics, Program for Cancer Biology and BIO-MAX Institute, Seoul National University, Seoul, South Korea; Nihon University School of Medicine, JAPAN

## Abstract

**Objective:**

Recent evidence has suggested that AHNAK expression is altered in obesity, although its role in adipose tissue development remains unclear. The objective of this study was to determine the molecular mechanism by which *Ahnak* influences adipogenesis and glucose homeostasis.

**Design:**

We investigated the *in vitro* role of AHNAK in adipogenesis using adipose-derived mesenchymal stem cells (ADSCs) and C3H10T1/2 cells. AHNAK-KO male mice were fed a high-fat diet (HFD; 60% calories from fat) and examined for glucose and insulin tolerances, for body fat compositions, and by hyperinsulinemic-euglycemic clamping. Energy expenditures were assessed using metabolic cages and by measuring the expression levels of genes involved in thermogenesis in white or brown adipose tissues.

**Results:**

Adipogenesis in ADSCs was impaired in AHNAK-KO mice. The loss of AHNAK led to decreased BMP4/SMAD1 signaling, resulting in the downregulation of key regulators of adipocyte differentiation (*P<*0.05). AHNAK directly interacted with SMAD1 on the *Ppar*γ*2* promoter. Concomitantly, HFD-fed AHNAK-KO mice displayed reduced hepatosteatosis and improved metabolic profiles, including improved glucose tolerance (*P*<0.001), enhanced insulin sensitivity (*P*<0.001), and increased energy expenditure (*P<*0.05), without undergoing alterations in food intake and physical activity.

**Conclusion:**

AHNAK plays a crucial role in body fat accumulation by regulating adipose tissue development via interaction with the SMAD1 protein and can be involved in metabolic homeostasis.

## Introduction

White adipose tissue (WAT) is important for maintaining energy balance, storing calories as lipids, and supplying energy sources following hormonal stimulation [1]. Recent findings suggest that blocking TGFβ/SMAD3 signaling protects mice from obesity and diabetes by regulating glucose and energy homeostasis [2,3]. Evidence suggests that BMPs and TGFβ-pathway members involved in adipocyte differentiation and the SMAD family play important roles in BMP/TGFβ signaling [4–6]. Recently, we reported that AHNAK regulates cell growth by mediating TGFβ/SMAD3 signaling [7].

AHNAK, located on human chromosome 11q12–13, encodes a ~700-kDa protein initially identified in human neuroblastomas and skin epithelial cells [8–10]. The AHNAK scaffolding protein regulates several signal-transduction pathways, including those related to cytoskeletal structure formation, calcium homeostasis, and muscle regeneration and calcium signaling with protein kinase C (PKC) and phospholipase Cγ [11–14]. We previously reported that AHNAK-KO mice are protected from diet-induced obesity, which was associated with differences in urine and blood metabolomic profiles between HFD-fed wild-type and AHNAK-KO mice [15]. Li *et al*. also showed that AHNAK was upregulated in adipose tissues of diet-induced-obesity rat models [16]. However, the molecular mechanism of the action of AHNAK during adipose tissue development remains unclear.

Here, we investigated the molecular mechanism whereby fat accumulation was reduced by impaired adipocyte development in AHNAK-KO mice, which had improved metabolic profiles. Therefore, this study demonstrated the role of Ahnak in adipogenesis and metabolic homeostasis through BMP4/SMAD1 signaling in adipocytes.

## Materials and Methods

### Experimental animals

All mice used in this study had a C57BL/6 background. AHNAK-KO mice were generated by disrupting exon 5 of the *Ahnak* gene, as described [17]. AHNAK-KO (*Ahnak* ^-/-^) mice were obtained by crossing heterozygous breeders. 6-week-old male KO and wild-type mice were fed either a regular chow (RC) diet or a high fat diet (HFD; 20% carbohydrate, 60% fat, 20% protein; D12492; Research Diets Inc., New Brunswick, NJ, USA). Mice were maintained under a 12-h light-dark cycle and had free access to water in a specific pathogen-free barrier facility. Mice were randomly assigned. These procedures were approved by the Institutional Animal Care and Use Committee of Seoul National University (Permit Number: SNU-201004-14) in accordance with the Principles of Laboratory Animal Care (NIH publication no. 85–23, revised 1985).

### Cell culture, reagents, and plasmids

Murine 3T3-L1 and C3H10T1/2 cells were kindly provided as a gift by Dr. Jae Woo Kim (Yonsei University, Seoul, South Korea) [18,19]. Cells were differentiated into mature adipocytes as described [18], with modifications. Flag-*Ahnak* (N-terminal, amino-acid residues 1–150) and HA-*Smad1* [20] expression vectors were purchased from GeneCopoeia (Rockville, MD, USA) and Addgene, respectively. C3H10T1/2 cells were transfected directly with total of 4 μg plasmid DNA using Lipofectamine LTX with Plus reagent (Invitrogen, Carlsbad, CA, USA) according to the manufacturer's protocol. FlexiTube small interfering RNA (siRNA) directed against murine *Ahnak* mRNA (accession no. NM_001039959) was from Qiagen. siRNA transfections were performed with Lipofectamine RNAiMAX (Invitrogen), according to the manufacturer’s protocol. AllStars Negative Control siRNA was obtained from Qiagen (Hilden, Germany).

### Isolation and expansion of adipose-derived stem cells (ADSCs)

The establishment of wild-type (WT) and AHNAK-KO ADSCs isolated from epididymal and inguinal WATs of 8–10-week-old mice was described previously [21,22]. Briefly, adipose tissue was minced and digested with 0.75 mg/ml collagenase (Wako Pure Chemical Industries, Ltd., Osaka, Japan) for 2 h at 37°C. Cells were added to RBC lysis buffer (eBioscience, San Diego, CA) and filtered through a 100-μm cell strainer (BD Falcon, Franklin Lakes, NJ, USA) to generate single-cell suspensions. Cells were diluted to 1 × 10^5^ cells/cm^2^ in DMEM/F12 medium (Gibco, pH 7.4) containing 10% FBS and maintained in 5% CO_2_ at 37°C.

### Quantitative real-time PCR (qPCR) analysis

Total RNA was isolated using the Total RNA Purification System (Invitrogen) following the manufacturer’s protocol. qPCR was performed with SYBR Green dye using an ABI Step One Real-Time PCR instrument (Applied Biosystems, Cheshire, U.K.). For relative quantification of gene expression, we used the ΔΔCt method. Results were normalized to expression of the control gene *36B4*. Primers were designed according to published complementary DNA or genomic sequences. The primer sequences are shown in S1 Table.

### Immunoblotting and immunoprecipitation experiments

For immunoblot analysis, cells were lysed in PRO-PREP buffer (iNtRON Biotechnology Inc., Seoul, Korea) containing a phosphatase-inhibitor cocktail (GenDEPOT, Barker, TX, USA). Protein extracts were separated by sodium dodecyl sulfate-polyacrylamide gel electrophoresis, transferred to a polyvinylidene fluoride membrane (Millipore, Billerica, Massachusetts, USA), and subjected to immunoblot analysis. Cytosolic and nuclear fractions used in immunoblot analysis were prepared using the NE-PER Nuclear and Cytoplasmic Extraction Kit (Thermo Scientific, Rockford, IL, USA), according to the manufacturer's instructions. For immunoprecipitation experiments, lysates were immunoprecipitated with an anti-HA antibody at 4°C overnight. Immunocomplexes were washed three times with lysis buffer (1% Triton X-100, 20 mM HEPES at pH 7.5, 150 mM NaCl, 12.5 mM, 10% glycerol, 5 mM EDTA, proteinase inhibitor cocktail [GenDEPOT], and a phosphatase inhibitor), mixed with 2X sample buffer, and separated from the protein A/G agarose beads (Santa Cruz) by boiling. Immunoblot analysis was performed using the indicated antibodies. Proteins were visualized by ECL chemiluminescence (AbClon, Seoul, Korea). GAPDH was detected as a loading control. Immunoreactive signals were detected through their enhanced chemiluminescence and recorded using the MicroChemi 4.2 system (DNR Bio-Imaging Systems, Jerusalem, Israel).

### Metabolic assays

For glucose-tolerance tests (GTTs), mice were fasted for 16 h and injected intraperitoneally with 1.5 mg of glucose/g of body weight. For insulin-tolerance tests (ITTs), mice were fasted for 6 h and injected intraperitoneally with insulin (Humulin R; Eli Lilly, Indianapolis, IN) at 1 unit/kg of body weight.

### Basal study

A comprehensive animal metabolic monitoring system (CLAMS; Columbus Instruments, Columbus, OH) was used to evaluate activity, food consumption, and energy expenditure before and after HFD feeding. Energy expenditure and food intake data were normalized with respect to lean body weight. Energy expenditure and RQ were calculated from the gas exchange data.

### Body composition

Fat and lean body masses were assessed by ^1^H magnetic resonance spectroscopy (Bruker BioSpin, Billerica, MA) before and after mice were subjected to an HFD.

### Immunofluorescence

ADSCs were grown on chamber slides and exposed to BMP4 for 1 hour. Cells were fixed and then permeabilized in 0.25% Triton-X 100 at room temperature for 10 min. After washing with PBS, the cells were blocked with 10% normal goat serum. For nuclear staining, the samples were incubated with DAPI (Vector Laboratories). Images were captured using a confocal microscope system (LSM 710, Carl Zeiss, Germany).

### Hyperinsulinemic-euglycemic clamp study

Seven days prior to performing hyperinsulinemic/euglycemic clamp studies, indwelling catheters were placed in the right internal jugular vein extending to the right atrium. To estimate insulin-stimulated whole-body glucose fluxes, [3-^3^H] glucose was infused throughout the clamps at a rate of 0.1 μCi/min, and 2-deoxy-D-[1-^14^C] glucose (2-[^14^C]DG; Perkin Elmer) was injected after 85 min following clamping to estimate the rate of insulin-stimulated tissue glucose uptake, as described [23].

### Glucose flux calculations

Whole-body glycolysis was calculated as the rate of increase in the plasma [^3^H]_2_O concentration divided by the specific activity of plasma [^3^H] glucose, as described [24]. Whole-body glycogen synthesis was estimated by subtracting whole-body glycolysis from whole-body glucose uptake, assuming that glycolysis and glycogen synthesis account for the majority of insulin-stimulated glucose uptake [25].

### Statistics

All values are expressed as the mean±SEM. Statistical analysis was performed using the Student’s t test between two groups. *P*<0.05 was considered statistically significant.

## Results

### Ahnak-KO mice are resistant to diet-induced obesity and had reduced adiposity

**Fig 1:**
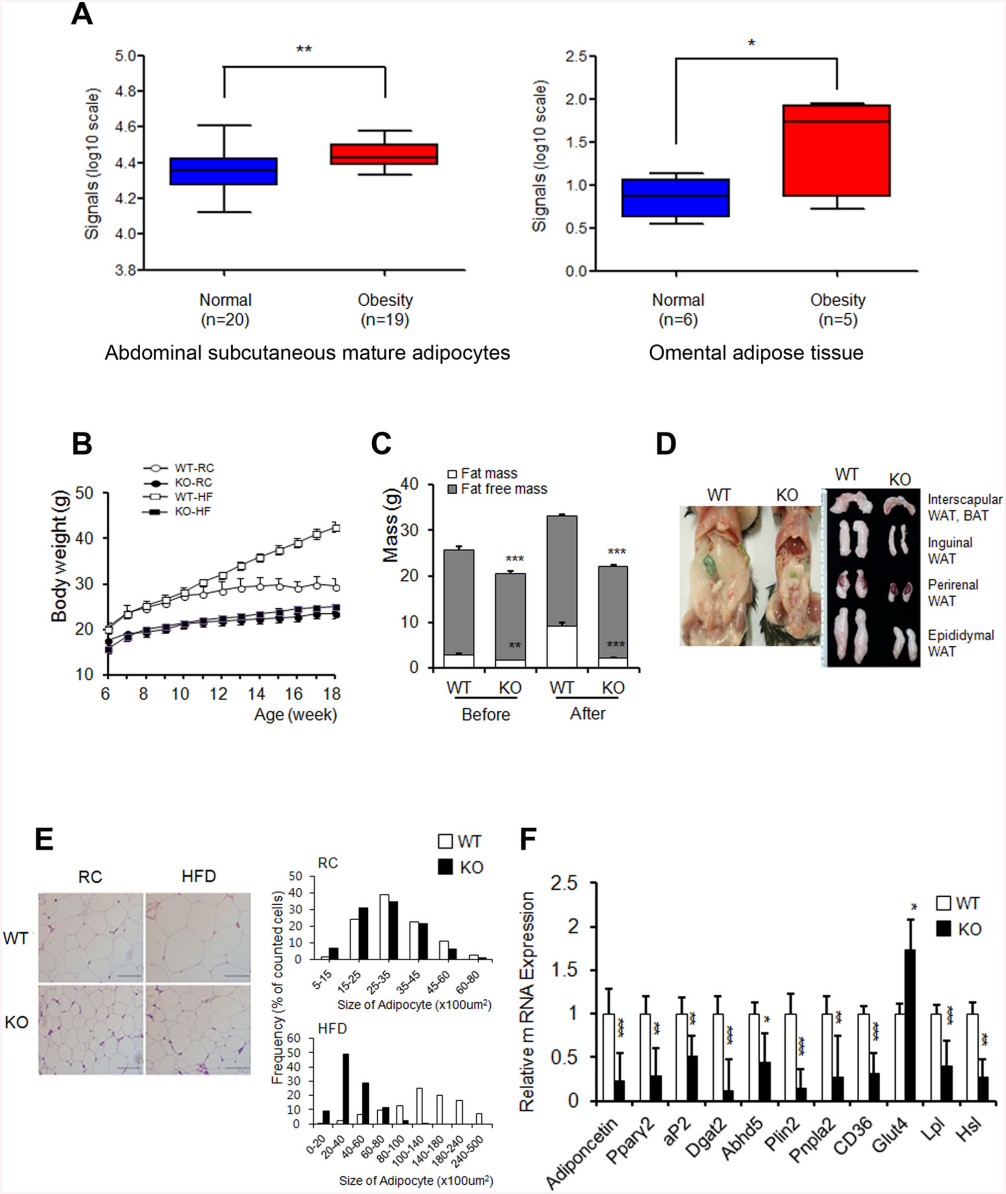
*Ahnak*-KO mice displayed reduced adiposity. (**A**) AHNAK expression of obese human subjects. (**B**) Changes in body weights during 12 weeks of feeding on RC or an HFD; *n* = 3 for WT and KO mice on regular chow diet (RC), *n* = 4 for WT and KO mice on HFD. (**C**) Body-composition changes in mice after 4 weeks on an HFD; WT: *n* = 6, KO: *n* = 5. (**D**) Representative pictures of fat pads from mice fed an HFD. (**E**) H&E staining (left) of eWAT and distribution of adipocyte sizes (right); *n* = 3 Scale bar, 200μm. (**F**) Relative mRNA levels of the indicated genes in WATs were measured by qPCR. Values were normalized to *36B4*. WT: *n* = 5, KO: *n* = 8 (**F**). The data shown are the mean±SEM. *P<0.05, **P<0.01, ***P<0.001 between WT and KO mice. eWAT, epididymal white adipose tissue.

To study the potential correlation between AHNAK expression and obesity in humans, we analyzed existing GEO data. AHNAK expression was significantly increased in both subcutaneous mature adipocytes (GDS 1495, *P*<0.01) and omental adipose tissues (GDS 3688, *P*<0.05) from obese subjects (Fig 1A). The tissue distribution of AHNAK was examined in C57BL/6 mice by qPCR analysis. *Ahnak* mRNA was abundantly expressed in eWAT, brown adipose tissue (BAT), heart tissue, and lung tissue (S1A Fig). To elucidate the physiological role of AHNAK *in vivo*, we maintained *Ahnak*-KO, *Ahnak* ^+/-^ (hetero) mice, and WT littermates. No differences in body weights were observed between WT and *Ahnak* ^+/-^ mice (S1B Fig). However, AHNAK-KO mice weighed less than WT mice at 6 weeks (S1C Fig). At 18 weeks of age, KO mice had gained markedly less weight, compared to the WT controls fed either RC or an HFD (Fig 1B and see also S1C Fig). We next analyzed the body composition of WT and KO mice. We observed a significant increase in the fat-to-body ratio in WT mice, but virtually no change in KO mice after an HFD (Fig 1C). Consistently, the major fat pads of KO mice were significantly smaller than those of WT mice (Fig 1D). Micro-CT results revealed that the total fat volume in the abdominal area of KO mice decreased (S1D Fig). Adipocytes in KO mice contained a higher frequency of small adipocytes and a lower frequency of mid- and large-sized adipocytes than their WT counterparts (Fig 1E).

In agreement, the expression of adipogenic-related genes such as *Ppar*γ*2*, a key regulator of adipogenesis; *aP2*, a late adipocyte differentiation marker; and adiponectin, were reduced in eWAT from HFD-fed KO mice compared to WT mice. Furthermore, the expression of genes related to lipid metabolism, including *Dgat2*, *Plin2*, *Cd36*, *Lpl*, and *Hsl* was significantly decreased in eWATs from HFD-fed KO mice (Fig 1F). In contrast, minor differences in adiponectin, *Lpl*, and *Hsl* expression occurred in RC-fed mice (S1E Fig).

Because KO mice displayed significantly decreased fat mass, we examined the liver as a secondary fat-storage organ. The ratios of liver-to-body weight between KO and WT mice were not significantly different, even though the KO livers were lighter than WT livers fed on either RC or an HFD (S2A Fig). Hepatic triglyceride and free fatty acid (FFA) levels decreased in KO mice (S2B Fig). In agreement, HFD-fed KO mice displayed reduced hepatic fat accumulation and protection against hepatosteatosis (S2C Fig). These findings indicated that AHNAK KO mice were protected from diet-induced obesity and fat accumulation, consistent with the reduced expression of lipogenic genes, which are not lipodystrophic.

### Requirement of Ahnak for adipocyte differentiation *in vitro*

**Fig 2:**
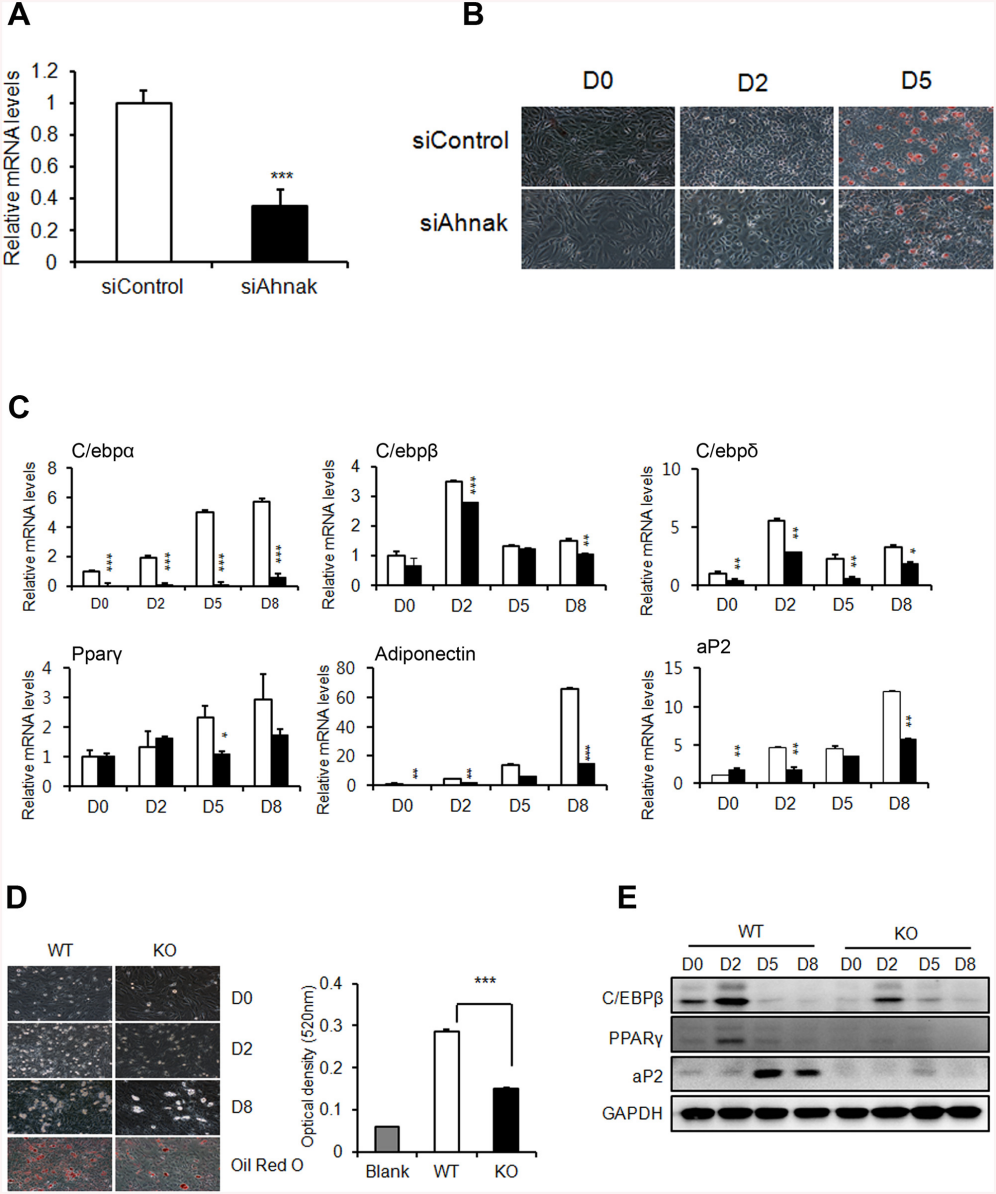
AHNAK plays a role in adipogenesis *in vitro*. (**A**) C3H10T1/2 cells were transfected with control siRNA and *Ahnak* siRNA. (**B**) C3H10T1/2 cells underwent adipocyte differentiation, as determined by oil red O staining. (**C**) Expression levels of adipogenic genes were measured by qPCR. All genes were normalized to *36B4*. (**D**) Representative pictures showing differentiated ADSCs stained with oil red O at day 8 after the induction of differentiation. (**E**) Immunoblot analysis of adipogenic proteins from differentiated cells. GAPDH was used as a loading control.

The *Ahnak* mRNA levels in normal (B6) and obese (Ob/Ob) mice markedly increased in the SVF of WAT, which contains multipotent mesenchymal stem cells (S3A Fig). AHNAK expression was also decreased during adipocyte differentiation in 3T3-L1 cells (S3B Fig), where fully differentiated cells contained mainly adipocytes. C3H10T1/2 cells transfected with *Ahnak*-specific siRNA, were stained with oil red O to examine lipid accumulation (Fig 2A and 2B). We found that the expression of *C/ebp* family members *Ppar*γ2, aP2, and adiponectin significantly decreased during adipogenesis following *Ahnak* silencing, relative to that observed in control-siRNA transfectants (Fig 2C). Consistently, ADSCs from KO mice showed significantly inhibited formation of lipid-containing adipocytes (Fig 2D). The adipogenic markers were significantly suppressed in AHNAK deficient ADSCs (Fig 2E). Formation of lipid-laden droplets was apparently reduced in fully differentiated 3T3-L1 cells by transfection with *Ahnak*-specific siRNA (S3C Fig). Thus, AHNAK expression in the SVF in WAT may explain the low efficiency of mature adipocyte differentiation observed in AHNAK-deficient mesenchymal stem cells and the reduced adiposity of AHNAK KO mice.

### AHNAK is required for SMAD1/5 activation, which is essential for BMP4-dependent adipogenic differentiation

**Fig 3:**
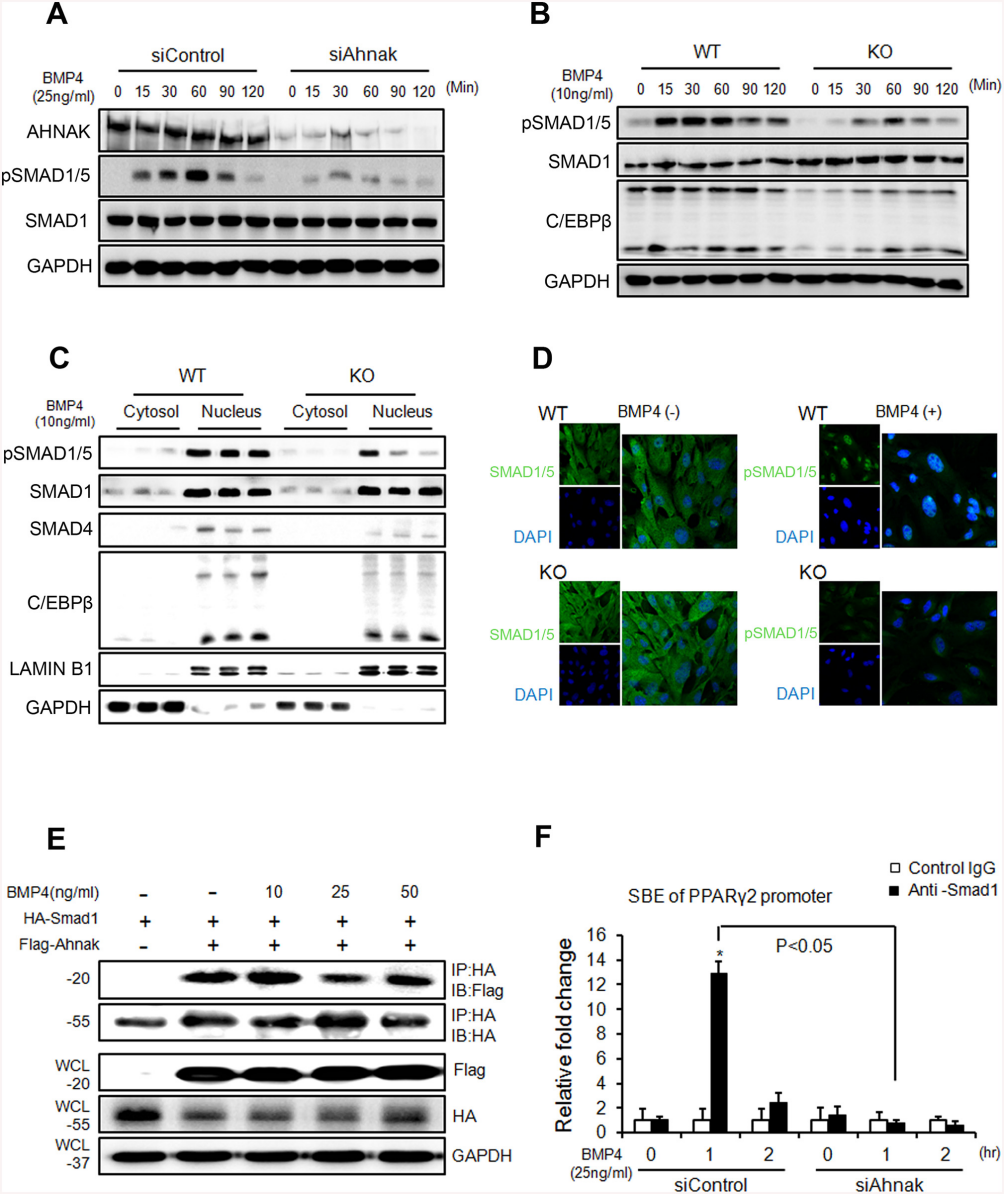
AHNAK is required for SMAD1/5 activation, which is essential for adipogenic effects caused by BMP4 signaling. (**A, B**) Following BMP4 stimulation of C3H10T1/2 cells (**A**) and ADSCs (**B**) for the indicated times, the levels of indicated proteins were measured by immunoblotting. (**C**) Following BMP4 treatment for 1 h, cell lysates from ADSCs were subjected to nuclear and cytoplasmic fractionation. Proteins from each fraction were studied by immunoblot analysis. LAMIN B1 and GAPDH were used as nuclear and cytoplasmic markers, respectively. (**D**) Immunofluorescence staining showing the subcellular localizations of SMAD1/5 in ADSCs. DAPI was used for nuclear staining. (**E**) C3H10T1/2 cells were transfected with plasmids expressing Flag-*Ahnak* and HA-*Smad1*, after which the cells were incubated without or with BMP4 for 60 min. (**F**) DNA-protein complexes from C3H10T1/2 cells were immunoprecipitated with an anti-SMAD1 antibody or a control IgG. Enrichment of the DNA fragment containing SMAD-binding sites on the *Ppar*γ*2* promoter was evaluated by qPCR.

BMP signaling induces the commitment of mesenchymal stem cells into preadipocytes [9,26]. SMAD1/5/8 phosphorylation is critical for BMP4-mediated transcriptional regulation and subsequent commitment to adipocyte differentiation^9^. Thus, we examined whether AHNAK is required for BMP4 signaling. Control C3H10T1/2 cells and ADSCs treated with recombinant BMP4 showed increased SMAD1/5 phosphorylation in a time-dependent manner (Fig 3A and 3B). However, in AHNAK-deficient C3H10T1/2 cells and ADSCs, BMP4-induced SMAD1/5 phosphorylation was repressed (Fig 3A and 3B). Importantly, nuclear pSMAD1/5 translocation occurred following BMP4 treatment in WT ADSCs, but not in KO ADSCs (Fig 3C and 3D). AHNAK protein is also translocated into nucleus following BMP4 stimulation (S4 Fig). In addition, the expression of SMAD4, a co-SMAD that binds to R-SMADs such as SMAD1/5/8 to form an active SMAD complex, was suppressed in AHNAK-KO ADSCs after BMP4 treatment, compared to that in WT ADSCs. The early adipogenic transcription factor C/EBPβ showed a similar trend in terms of expression (Fig 3C). Our data suggested that the AHNAK protein potentiates BMP4 signaling by interacting with SMAD1. To study this potential interaction, Flag-*Ahnak* and HA-*Smad1* expression vectors were co-transfected into C3H10T1/2 cells and subjected to co-immunoprecipitation with an anti-HA antibody. The transfection of Flag-*Ahnak* expressed vector had no effect on cell viability (S5A Fig). These results indicated that AHNAK interacted with SMAD1 in a BMP4-independent manner (Fig 3E). Moreover, a proximity ligation assay (PLA) using AHNAK and SMAD1 probes showed that AHNAK-SMAD1 complexes were detected as fluorescent spots, both in BMP4-treated and vehicle-control cells (S5B Fig). BMP2/4-SMAD signaling induced adipogenic commitment, which regulates PPARγ expression [5,27]. Importantly, Ahnak deficiency causes decreased PPARγ2 expression (Figs 1 and 2). We thus evaluated the effects of AHNAK on SMAD1-dependent *Pparγ2* transcriptional regulation using a chromatin immunoprecipitation (ChIP) assay. The ChIP complex detected with an anti-SMAD1 antibody revealed significantly increased association with the Smad-binding element (SBE) in the *Pparγ2* promoter following BMP4 treatment in control siRNA-transfectants. However, *Ahnak*-downregulated cells did not exhibit a SMAD1-bound *Ppar*γ*2* promoter DNA fragment (Fig 3F). These results indicate that AHNAK is required for SMAD1 activation and SMAD1 binding to the *Pparγ2* promoter.

### *Ahnak*-KO mice exhibit enhanced glucose tolerance and increased systemic insulin sensitivity

**Fig 4:**
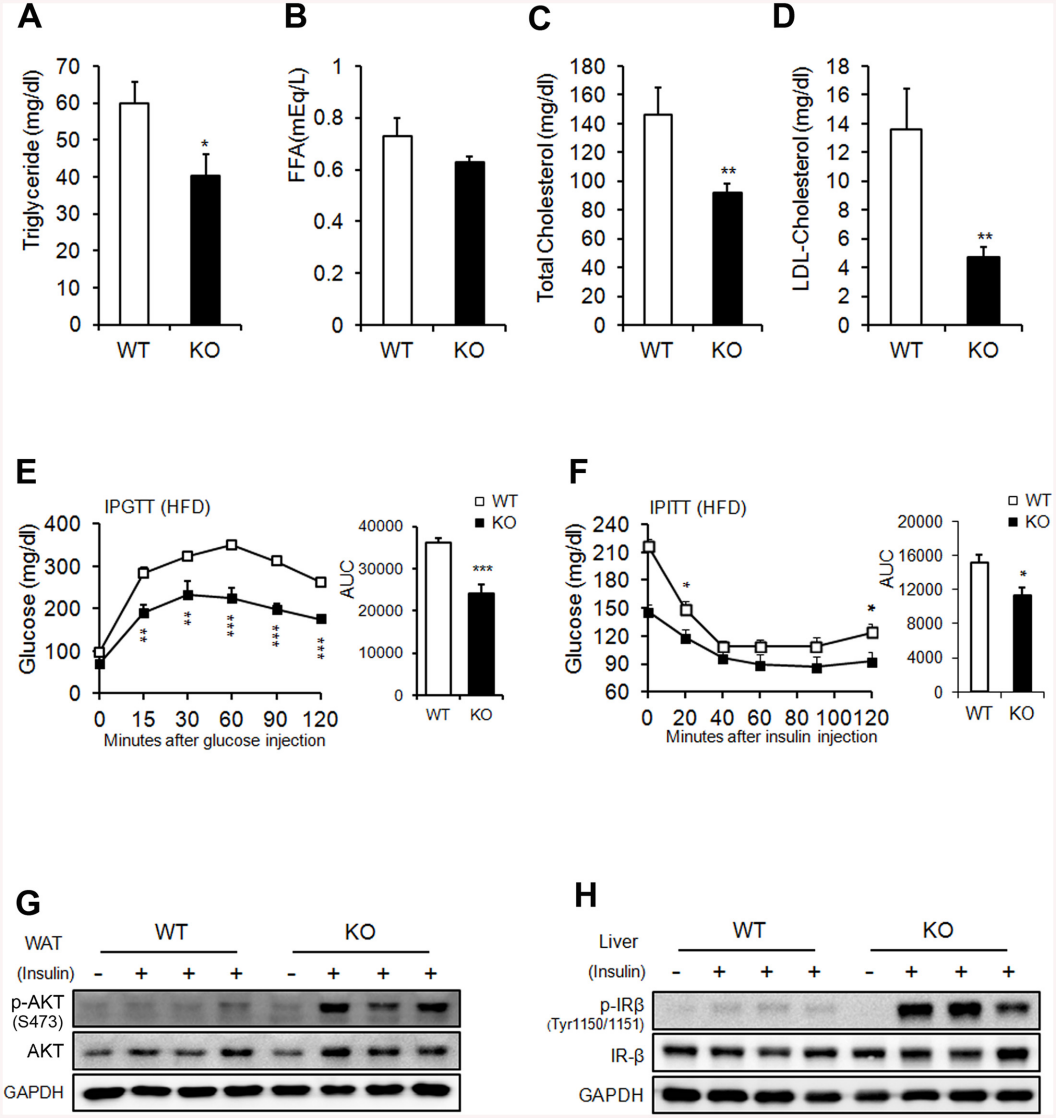
Metabolic parameters. (**A**) Serum triglyceride. (**B**) FFA. (**C**) Total cholesterol. (**D**) LDL-cholesterol in WT and KO mice on an HFD. Serum levels were measured after a 16-h fasting period; *n* = 9/group (a-d). (**E**) Glucose tolerance after an 8-week HFD; *n* = 9 for WT and n = 6 for KO mice. (**F**) Insulin tolerance after an 8-week HFD; *n* = 6 for WT and n = 6 for KO mice. (**G, H**) Insulin signaling pathway in WAT (**G**) and liver (**H**). The data shown are the mean±SEM. *P<0.05, **P<0.01, ***P<0.001 between WT and KO mice.

To assess the systemic effect of reduced adiposity in AHNAK-KO mice, we measured lipid-metabolism parameters in serum. The triglyceride levels were reduced in KO mice, although the FFA levels remained unchanged (Fig 4A and 4B). The levels of total and LDL-cholesterol were substantially decreased in HFD-fed KO mice (Fig 4C and 4D). Next, we performed intraperitoneal GTT to determine the ability to clear glucose, with blood samples from RC- and HFD-fed mice. KO mice on an HFD displayed lower blood glucose levels under fasting conditions and after glucose administration (Fig 4E) than did WT mice. ITTs revealed that the blood-glucose lowering effect of insulin was slightly better in KO mice (Fig 4F). Glucose tolerance and insulin sensitivity were not significantly different in RC-fed mice (S6A and S6B Fig). Key mediators of insulin signaling were examined to clarify the mechanisms of improved glucose metabolism in KO mice. The phospho-AKT levels in WAT, BAT, and gastrocnemius muscle, and phospho-IRb levels in liver increased in HFD-fed KO mice after insulin stimulation (Fig 4G and 4H and see also S6C and S6D Fig). Interestingly, Glut4 mRNA was increased in eWAT from HFD-fed KO mice (Fig 1F). These results are consistent with previous studies that overexpression of human GLUT4 prevented the obesity-induced hyperinsulinemia and downregulation of GLUT4 in adipose tissue caused insulin resistance in mice [28,29]. Nevertheless, int remains to be investigated whether Ahnak regulates translocation of GLUT4 to plasma membrane upon insulin stimulation. Taken together, these results indicated that AHNAK-deficiency caused reduced adiposity and increased glucose tolerance and insulin sensitivity.

**Fig 5:**
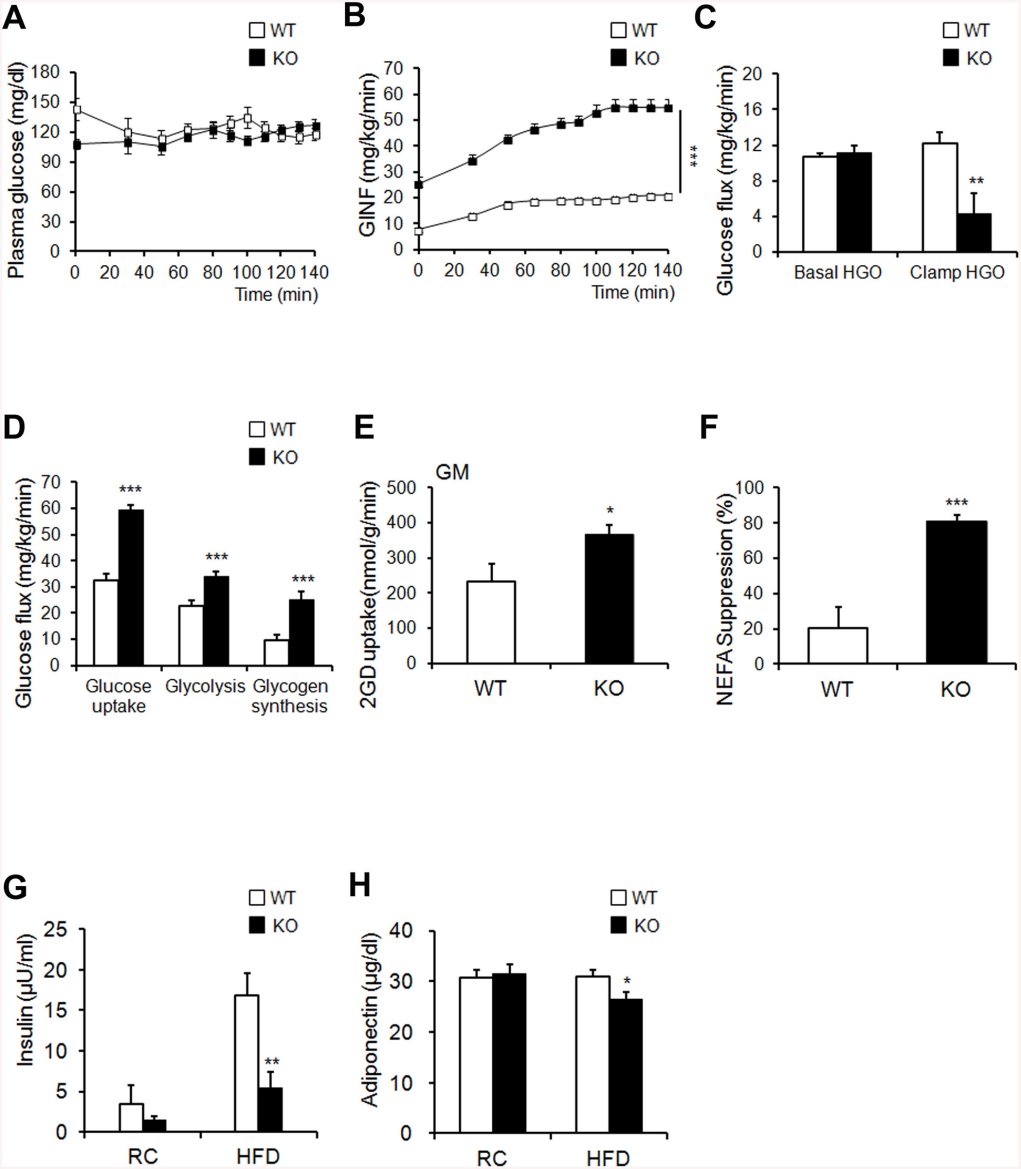
*Ahnak* KO mice exhibit enhanced glucose tolerance and insulin sensitivity. (**A, B**) Blood glucose levels during the hyperinsulinemic-euglycemic clamp. Glucose levels required to maintained euglycemia in both mice (**A**). Glucose infusion rate (GINF) during clamp study *Ahnak*-KO mice exhibited enhanced: the clamp study (**B**). (**C**) Insulin-mediated suppression of hepatic glucose output. (**D**) Whole-body glucose flux calculation (peripheral insulin sensitivity). (**E**) Insulin-stimulated 2-DG uptake in gastrocnemius muscle. (**F**) Suppression of plasma FFA levels during the clamp procedure; n = 7/group (**A–F**) (**G**) Serum insulin levels; *n* = 6 for WT and n = 5 for KO mice on RC, *n* = 9 for WT and KO mice on HFD. (**H**) Serum adiponectin levels; *n* = 9 for WT and *n* = 8 for KO mice on RC, *n* = 7 for WT and *n* = 9 for KO mice on HFD. The data shown are the mean±SEM. *P<0.05, **P<0.01, ***P<0.001 between WT and KO mice.

Next, we utilized a hyperinsulinemic/euglycemic clamp, enabling accurate determinations of insulin-dependent glucose uptake effects *in vivo*. First, during hyperinsulinemia, the glucose-infusion rate necessary to maintain euglycemia was higher in KO mice than in WT mice, indicating that insulin-stimulated glucose uptake and metabolism were active in KO mice (Fig 5A and 5B). Second, we tested whether hyperglycemia could result from elevated liver gluconeogenesis. Hepatic glucose output was lower in KO mice than in the WT control in the clamped condition (Fig 5C). The third possible explanation for hyperglycemia is decreased insulin-dependent glucose uptake into peripheral tissues. The glucose uptake, glycolysis, and glycogen synthesis were markedly higher in KO mice than in WT mice (Fig 5D). Insulin-stimulated 2-DG uptake in skeletal muscle was significantly increased in KO mice (Fig 5E). FFA suppression increased after insulin stimulation in KO mice (Fig 5F). The fasting insulin levels of HFD-fed KO mice were significantly decreased (Fig 5G). Thus, improved insulin sensitivity caused suppression circulating FFA levels in KO mice during clamp condition. Changes in adipokine secretion are correlated with several metabolic disorders [30]. Unexpectedly, serum adiponectin, which is inversely correlated with adipose tissue mass, was decreased in KO mice (Fig 5H). Collectively, these results indicate that KO mice showed enhanced peripheral insulin sensitivity and increased glucose metabolism, with activated insulin signaling cascades in the liver, muscle tissue, and adipose tissue, particularly when challenged by an HFD.

### AHNAK-KO mice show increased energy expenditure

**Fig 6:**
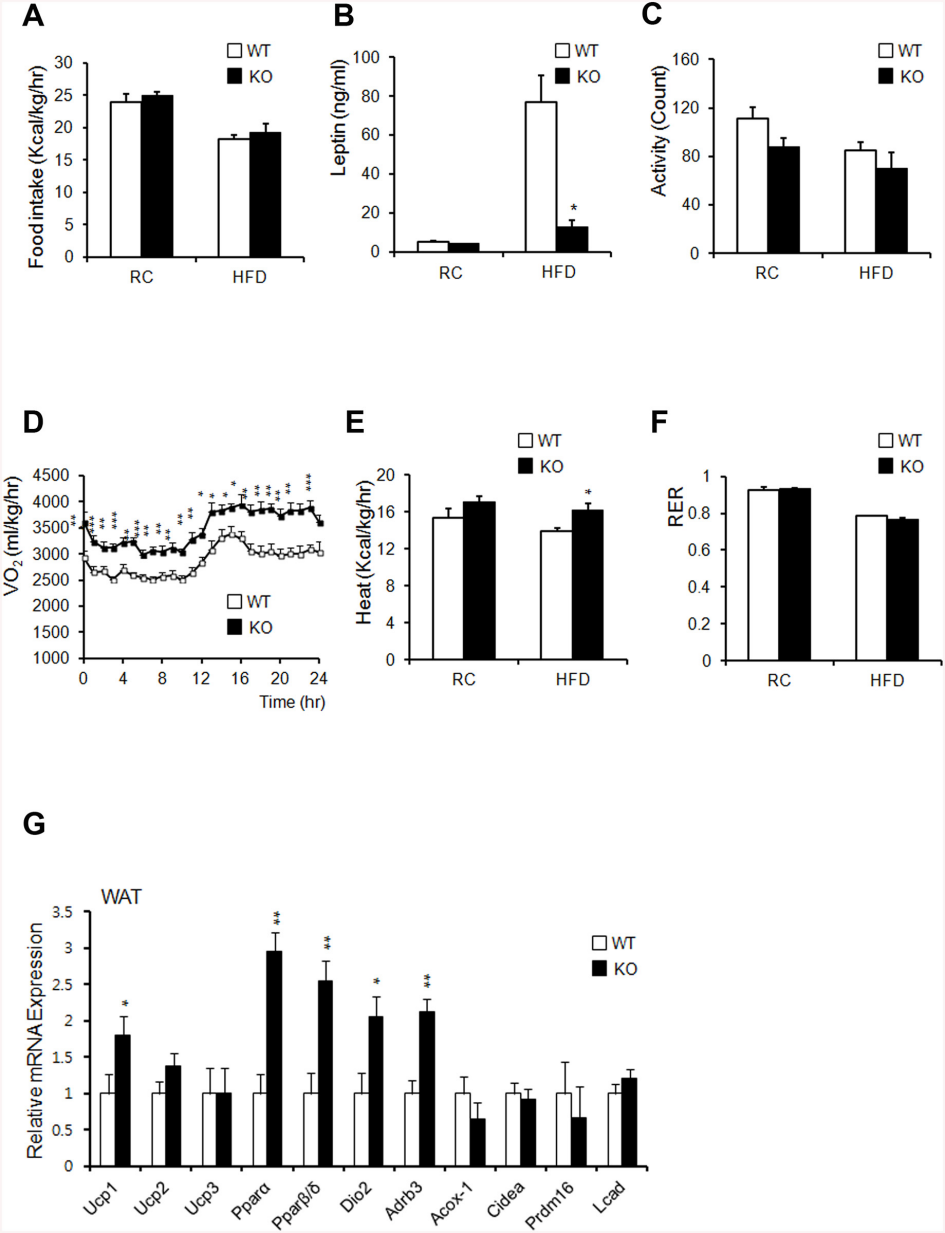
*Ahnak*-KO mice display increased energy expenditure. (**A**) Food intake (WT: *n* = 6, KO: *n* = 5). (**B**) Serum leptin concentrations (n = 4). (**C**) Measurement of locomotor activity (WT: *n* = 6, KO: *n* = 5). (**D**) Whole-body oxygen consumption (VO_2_) over the course of 24 h (WT: *n* = 6, KO: *n* = 5). (**E**) Average value of heat generation (WT: *n* = 6, KO: *n* = 5). (**F**) The respiratory exchange rate was calculated as CO_2_ production/O_2_ consumption (WT: *n* = 6, KO: *n* = 5). (**G**) Relative mRNA expression of genes involved in energy dissipation and in brown adipose-specific genes in WAT, as measured by qPCR (*n* = 6). Values were normalized to *36B4* expression. The data shown are the mean±SEM; *P<0.05, **P<0.01, ***P<0.001.

Changes in body weights often correlate with energy-balance alterations. The amount of calorie uptake/h in both RC- and HFD-fed mice was unaltered when normalized to body weight (Fig 6A). Serum leptin levels decreased in HFD-fed KO mice, despite their similar food intake (Fig 6B). These data suggested that leptin sensitivity was enhanced in KO mice and that the reduction in leptin levels in KO mice resulted from a reduction in fat mass, as leptin levels are associated with fat mass [31]. Despite similar food intake levels between the two groups, KO mice showed greater leanness and resistance to HFD-induced obesity (Fig 1B). We thus hypothesized that KO mice have an increased metabolic rate or physical-activity rate. KO mice showed a slightly decreased level of activity (Fig 6C). Oxygen consumption and heat generation in KO mice were significantly increased compared to those of WT controls (Fig 5D and 5E). The RER remained unchanged in both groups (Fig 5F).

To confirm the effect of AHNAK ablation in WAT and BAT, we analyzed thermogenic gene expression profiles by qPCR. We observed a significant increase in the expression of *Pparα* and *Pparβ/δ*. We also detected increased expression of *Ucp1*, *Dio2*, and *Adrb3* in WAT from HFD-fed KO mice (Fig 5G). These results suggested that uncoupled respiration and oxidation may contribute to increased oxygen consumption, which could cause resistance to diet-induced obesity in KO mice. Histologic evaluation of BAT displayed slightly reduced lipid-droplet accumulation in KO mice, compared to that in WT mice fed either RC or an HFD (S7A Fig). However, the BAT weights between the two groups were comparable (S7B Fig). The expression of *Ucp3*, *Pparα*, *Pparβ/δ*, *Acox-1*, and *Dio2* increased despite the unaffected levels of *Ucp1* in BAT of KO mice, compared to that of WT mice (S7C Fig). These results suggested that increased energy expenditure resulting from mitochondrial uncoupling in WAT of KO mice contributed to the resistance to diet-induced obesity.

## Discussion

The physiological functions of AHNAK in adipose tissues have been unclear, although AHNAK is highly expressed in WAT (S1A Fig). Here, we provide evidence that AHNAK acts as a critical regulator of adipogenesis *in vitro* and *in vivo*. Studies on the role of Ahnak in adipose tissue have reported conflicting results. Alli and coworkers claimed that decreased AHNAK1 expression observed during adipocyte differentiation in 3T3-L1 cells indicated that AHNAK negatively regulates adipogenesis [32]. In contrast, *Ahnak* gene expression was increased 6-fold in adipose tissue during early-phase obesity in rats [16]. Previous findings have demonstrated elevated AHNAK levels in WAT from mice with genetically or environmentally induced obesity [16,33]. Consistent with previous murine-model studies, AHNAK expression significantly increased in the fat tissues of obese human subjects (Fig 1A).

**Fig 7:**
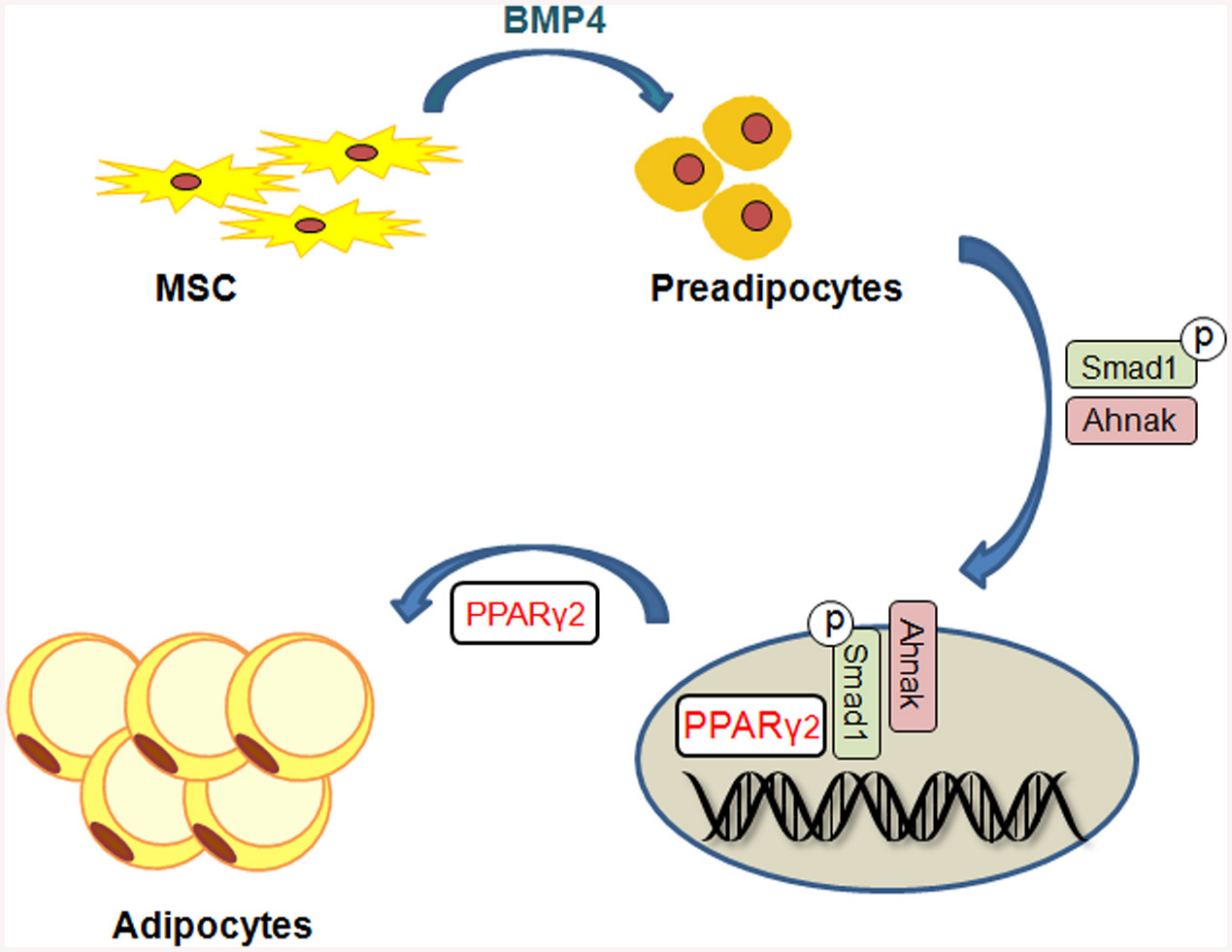
Putative model for the role of *Ahnak* in Smad1-dependent induction of PPARg2 by BMP signaling.

Loss of Ahnak resulted in lowered fat-cell formation due to suppressed SMAD1/5 phosphorylation and nuclear translocation. Our results indicated that AHNAK regulates adipocyte differentiation by modulating BMP-induced canonical SMAD signaling. SMAD1 inactivation in AHNAK-deficient ADSCs led to impaired *Pparγ2* transcription. Our data indicate that AHNAK is required for adipocyte differentiation via SMAD1 binding to the *Ppar*γ*2* promoter following BMP4 stimulation (Fig 7). Similar to AHNAK, SCHNURRI-2 interacts with SMAD1 and controls adipogenesis through *Pparγ2* transcriptional activation [34]. However, the possibilities underlying AHNAK involvement in regulation of the nuclear translocation or stability of pSMAD1 remain to be determined. Although additional studies are also required to elucidate the function of AHNAK-dependent SMAD1 signaling in adipose hypertrophy *in vivo*, previous evidence has shown that BMP/SMAD signaling promotes adiposity by Tob2 expression in mice [35].

Adipose tissue is composed of adipocytes and the stromal vascular fraction, which contains preadipocytes, mesenchymal stem cells, endothelial cells, and macrophages [36]. Adipocyte precursor cells in the stromal vascular fraction are capable of differentiation into mature adipocytes [37]. BMPs have crucial roles in cell fate-determinations of multipotent mesenchymal stem cells [38]. BMPs regulate gene expression of *Runx2*, *Pparγ2*, *Sox9*, and *MyoD*, which are master regulators of lineage determination that control embryogenesis and organogenesis [39–44]. Together, these findings imply that AHNAK present in the stromal vascular fraction of adipose tissue may explain the decreased AHNAK levels observed during adipogenesis, as well as the restricted adipogenic commitment and differentiation in AHNAK-KO mice. However, it remains unclear how AHNAK controls BMP4/SMAD1 signaling during mesenchymal differentiation into other cell types, such as the osteogenic, chondrogenic, and myogenic lineages. Furthermore, it would be interesting to determine whether AHNAK regulates BMP4 signal transduction via other inhibitory mechanisms such as BMP receptors, I-SMAD (SMAD6/SMAD7), or ubiquitination.

Previous studies reported that AHNAK activates PLC-γ1-PKCa and contributes to mitogenic signaling in response to growth factors [12,17]. The AHNAK protein controls cell cycle progression through c-MYC, which is highly expressed in AHNAK -deficient MEF cells [7]. Cell proliferation, arrest, cell-cycle re-entry, and differentiation by hormonal stimulation are crucial processes during adipogenesis [45]. Consistently, increased levels of c-MYC lead to the inhibition of adipocyte differentiation by blocking cell cycle arrest in 3T3-L1 cells [46,47]. Therefore, it would be interesting to uncover the link between AHNAK and c-MYC in 3T3-L1 cells, which are committed to an adipogenic lineage.

While we focused on the role of AHNAK in adipogenesis, our data also implicate AHNAK in energy and glucose homeostasis. Recent evidence showed that AHNAK expression inversely correlates with oxygen consumption [48]. Whole-body oxygen consumption and energy expenditure were significantly elevated in HFD-fed KO mice accompanied by increased levels of thermogenesis-related genes (*Ucp1*, *Dio2*, and *Adrb3*) and oxidation-related genes (*Pparα* and *Pparβ/δ*) in WAT, but no difference was found in BAT, which mainly controls heat generation. Adiposity influences systemic insulin resistance [49]. Here, we demonstrated that AHNAK KO mice are protected against HFD-induced insulin resistance, with reduced fat accumulation. A recent study showed that mice lacking AHNAK were glucose intolerant [33], and we also found that HFD-fed AHNAK-KO mice displayed markedly lower fasting insulin levels (Fig 5G). This reduction suggests that the increased glucose tolerance in AHNAK-KO mice was attributable to insulin sensitivity. Furthermore, our hyperinsulinemic/euglycemic clamp experiments revealed that HFD-fed AHNAK-KO mice displayed increased insulin-mediated suppression of hepatic glucose output, with higher glucose uptake in the peripheral tissues. In this regard, AHNAK deficiency also resulted in significantly improved glucose-disposal rates with a robust increase in insulin signaling in target tissues. These results may have been due to a difference in the fat contents of the diets. The authors performed their experiments with a lower fat-containing diet than was employed in the current study. Another possibility is that the mice had a different genetic background, which can influence metabolism in mice [50]. While our mutant mice were derived from a C57BL/6 background, their mice originated from BDF1, a cross between female C57BL/6 and male DBA/2. Because BDF1 mice are prone to develop obesity-induced diabetes more than B6 mice [51], our mutant mice could display better glucose tolerance and greater responsiveness to insulin.

Results from this study demonstrated that AHNAK downregulation protects mice from obesity, hepatosteatosis, and insulin resistance. Furthermore, although AHNAK is implicated in the regulation of energy metabolism and glucose homeostasis, further studies are needed to determine the molecular mechanism whereby AHNAK regulates energy metabolism. We cannot exclude the possibility that AHNAK may influence other tissues or mouse development because we used mice carrying the null mutation. In particular, studying tissue-specific AHNAK inactivation will likely be useful in that indirect effects may be involved in the overall regulation of adiposity and energy metabolism. Moreover, it should be determined whether AHNAK overexpression contributes to aggravated obesity and metabolic homeostasis.

Collectively, our data show that AHNAK promotes adipose tissue development and obesity by potentiating BMP4/SMAD1 signaling and that AHNAK can serve as a novel regulator of metabolic homeostasis. Our data thus expand the current knowledge regarding the early determination of adipose tissue development and highlight a potential indication for treating obesity-associated metabolic disorders.

## Supporting Information

S1 Fig Physiological characteristics of *Ahnak* KO mice. (**A**) Ahnak expression in various tissues from 6-week-old C57BL6 mice (n = 4–6). Values were normalized to Gapdh. (**B**) Comparison of body weights from wild type (*Ahnak* ^+/+^) and hetero (*Ahnak* ^+/-^) mice (n = 3). (**C**) Body weights of WT and KO mice at various ages. (**D**) Representative pictures of micro-CT showing abdominal cross-sections, where the white arrows indicate fat. (**E**) Relative mRNA levels of the indicated genes in WAT of RC-fed mice were measured by qPCR. Values were normalized to *36B4*. The data shown are the mean±SEM, *P<0.05, **P<0.01, ***P<0.001.
(TIF)

S2 Fig *Ahnak* KO mice are protected from hepatic steatosis-induced by an HFD. (**A**) Quantification of liver weights. (**B**) Quantification of liver triglycerides and FFAs in mice. (**C**) Representative pictures of H&E and oil red O staining of liver sections. The data shown are mean±SEM, n = 3–5.*P<0.05, **P<0.01, ***P<0.001 between WT and KO mice.
(TIF)

S3 Fig Loss of Ahnak inhibits adipocyte differentiation *in vitro*. (**A**) mRNA expression of Ahnak in the adipocyte fraction (Ad) and stromal vascular fraction (SVF) of eWATs (n = 4). (**B**) mRNA expression of Ahnak during adipogenesis in 3T3-L1 cells. (**C**) 3T3-L1 cells were transfected with siRNA before differentiation induction and subsequently stained with oil red O.
(TIF)

S4 Fig The subcellular localizations of Ahnak in C3H10T cells. DAPI indicates nuclear staining.
(TIF)

S5 Fig Interaction between Ahnak and Smad1. (**A**) Cell viability following transfection of Flag Ahnak expressed vector in C3H10T cells. (**B**) The Ahnak-Smad1 complexes were detected as spots in situ Duolink PLA. Nuclear material was stained by DAPI. The data shown are the mean±SEM, *P<0.05, **P<0.01, ***P<0.001 vs Mock.
(TIF)

S6 Fig Insulin signaling. (**A**) Glucose tolerance after feeding on RC; WT: *n* = 6, KO: *n* = 4. (**B**) Insulin tolerance after of feeding on RC; WT: *n* = 4, KO: *n* = 6. The data shown are the mean±SEM, *P<0.05, **P<0.01, ***P<0.001 between WT and KO mice. (**C** and **D**) Insulin signaling pathway in BAT (**C**) and gastrocnemius muscle (GM) (**D**) after an 8-week HFD.
(TIF)

S7 Fig Phenotypic characteristics of BAT. (**A**) H&E staining of BAT. Scale bar, 200 μm; (**B**) Quantification of BAT mass (left) and BAT mass normalized by body mass (right); WT: *n* = 5, KO: n = 4. (**C**) Relative mRNA expression involved in energy dissipation and brown adipose specific genes in BAT measured by qPCR from mice fed an HFD for 12 weeks (n = 6). Values were normalized to *36B4* expression. The data shown are mean±SEM; *P<0.05, **P<0.01, ***P<0.001.
(TIF)

S8 Fig Full unedited blots used in figures. Prestained Protein Marker (GenDEPOT, TX, USA) was used as a size marker. Therefore it is not visible in images obtained by CCD camera.
(TIF)

S1 Table Sequences of primers used for real-time PCR. (DOCX)
