# Correction: Obesity Resistance and Enhanced Insulin Sensitivity in *Ahnak* ^-/-^ Mice Fed a High Fat Diet Are Related to Impaired Adipogenesis and Increased Energy Expenditure

**DOI:** 10.1371/journal.pone.0144478

**Published:** 2015-12-02

**Authors:** Jae Hoon Shin, Il Yong Kim, Yo Na Kim, Sun Mee Shin, Kyung Jin Roh, Seo Hyun Lee, Mira Sohn, Soo Young Cho, Sang Hyuk Lee, Chang-Yong Ko, Han-Sung Kim, Cheol Soo Choi, Yun Soo Bae, Je Kyung Seong

The first sentence of the sub-section titled “AHNAK is required for SMAD1/5 activation, which is essential for BMP4-dependent adipogenic differentiation” in the Results section should have cited references 5 and 26 instead of 9 and 26.

The second sentence of the sub-section titled “AHNAK is required for SMAD1/5 activation, which is essential for BMP4-dependent adipogenic differentiation” in the Results section should not contain a superscripted 9 at the end of the sentence.

The eighth and ninth sentences of the first paragraph and the third sentence of the second paragraph of the sub-section titled “AHNAK-KO mice show increased energy expenditure” in the Results section should refer to Fig 6D and 6E, Fig 6F, and Fig 6G, respectively, instead of Fig 5D and 5E, Fig 5F, and Fig 5G.
