# Supplementary figures and images for: Obesity Resistance and Enhanced Insulin Sensitivity in Ahnak -/- Mice Fed a High Fat Diet Are Related to Impaired Adipogenesis and Increased Energy Expenditure
Source: PLoS One. 2015 Oct 14;10(10):e0139720. doi: 10.1371/journal.pone.0139720 (PMC4605776; doi:10.1371/journal.pone.0139720)

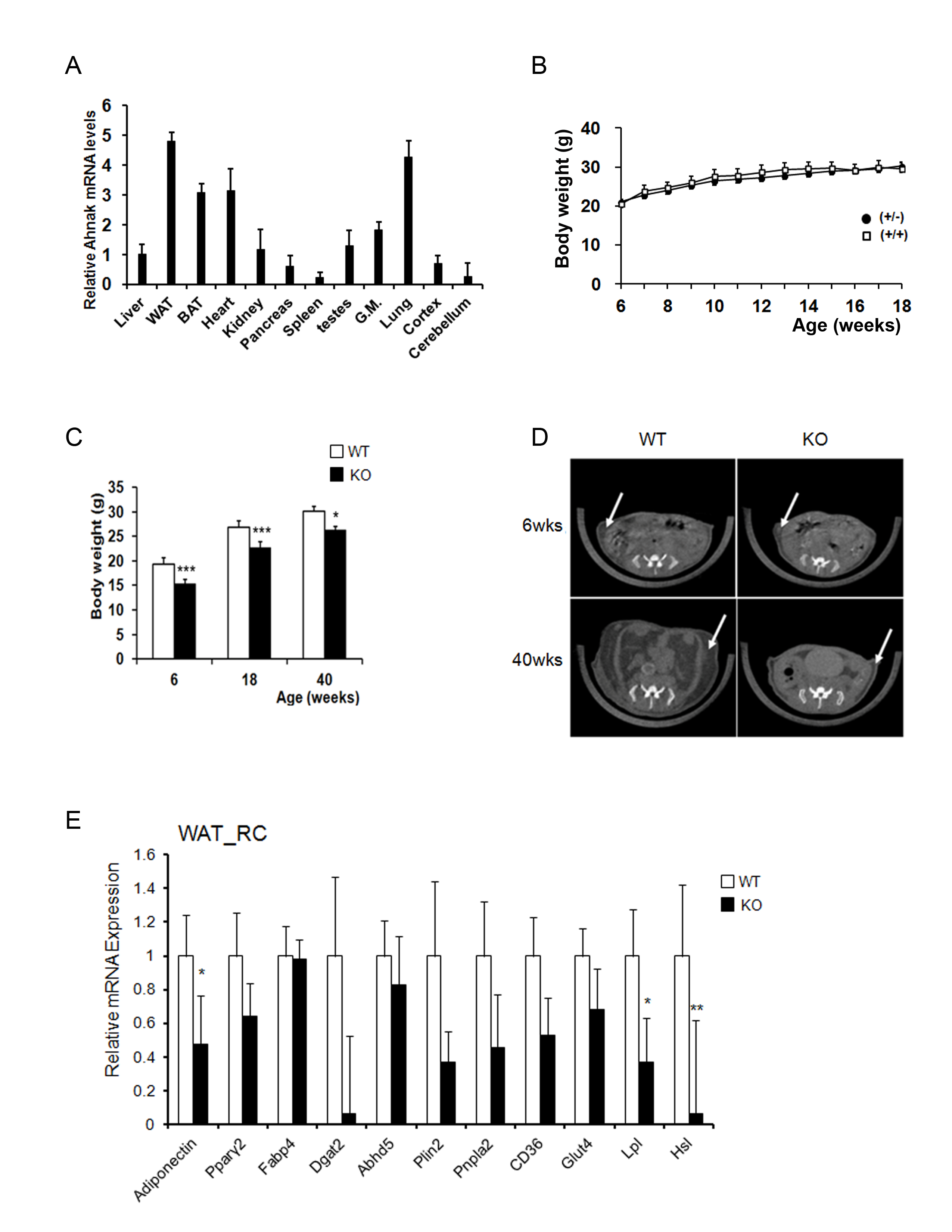

Supplement: S1 Fig — (A) Ahnak expression in various tissues from 6-week-old C57BL6 mice (n = 4–6). Values were normalized to Gapdh. (B) Comparison of body weights from wild type (Ahnak +/+) and hetero (Ahnak +/-) mice (n = 3). (C) Body weights of WT and KO mice at various ages. (D) Representative pictures of micro-CT showing abdominal cross-sections, where the white arrows indicate fat. (E) Relative mRNA levels of the indicated genes in WAT of RC-fed mice were measured by qPCR. Values were normalized to 36B4. The data shown are the mean±SEM, *P<0.05, **P<0.01, ***P<0.001. (TIF) [file pone.0139720.s001.tif]

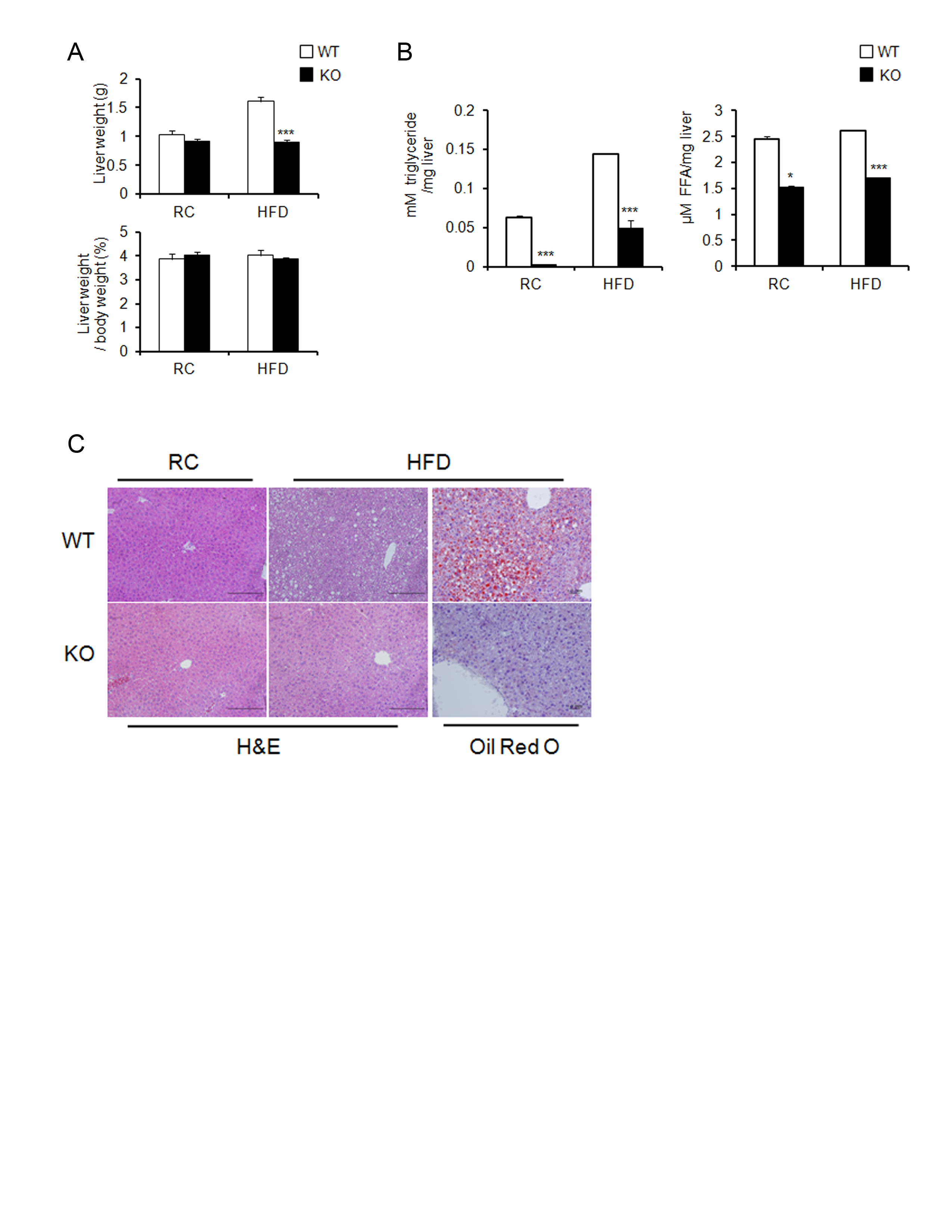

Supplement: S2 Fig — (A) Quantification of liver weights. (B) Quantification of liver triglycerides and FFAs in mice. (C) Representative pictures of H&E and oil red O staining of liver sections. The data shown are mean±SEM, n = 3–5.*P<0.05, **P<0.01, ***P<0.001 between WT and KO mice. (TIF) [file pone.0139720.s002.tif]

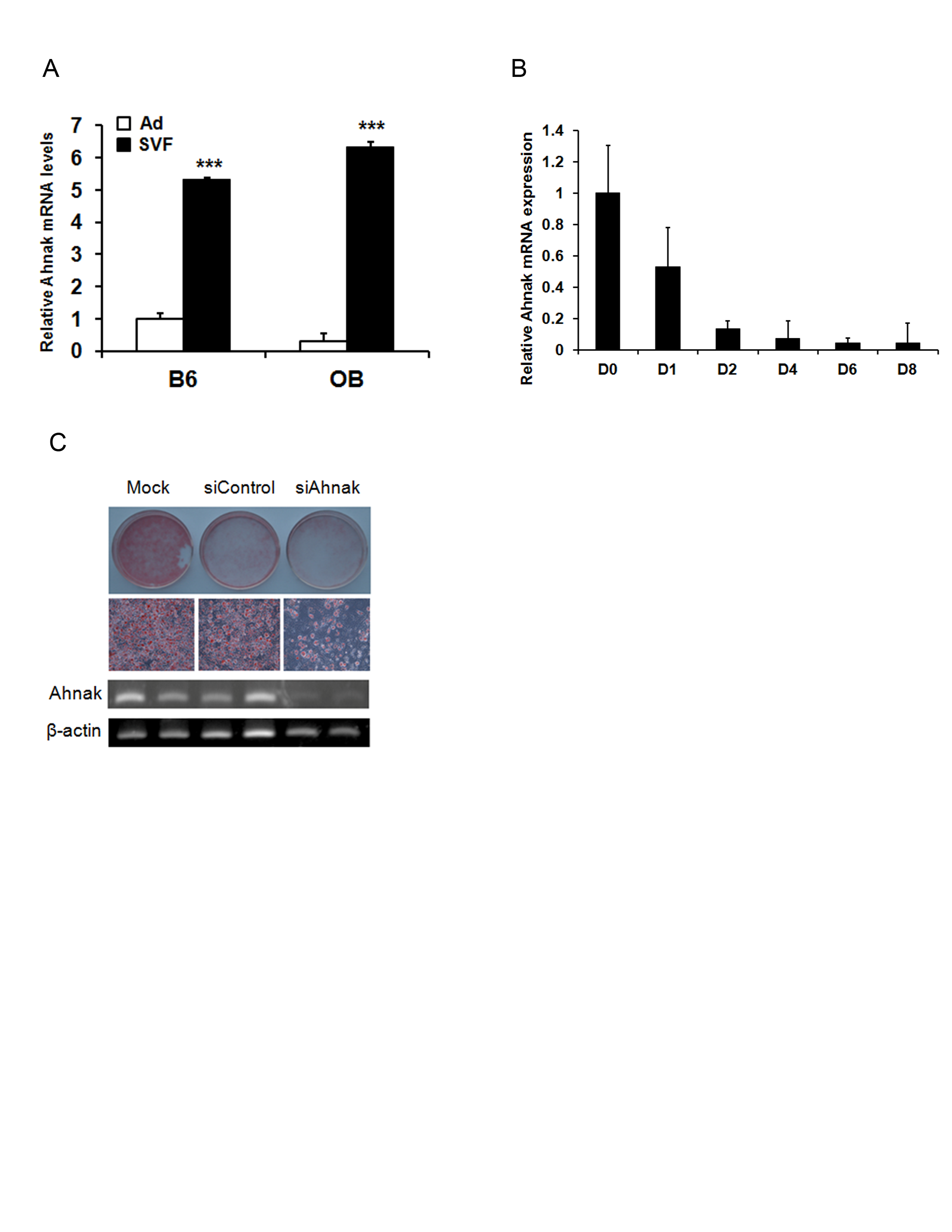

Supplement: S3 Fig — (A) mRNA expression of Ahnak in the adipocyte fraction (Ad) and stromal vascular fraction (SVF) of eWATs (n = 4). (B) mRNA expression of Ahnak during adipogenesis in 3T3-L1 cells. (C) 3T3-L1 cells were transfected with siRNA before differentiation induction and subsequently stained with oil red O. (TIF) [file pone.0139720.s003.tif]

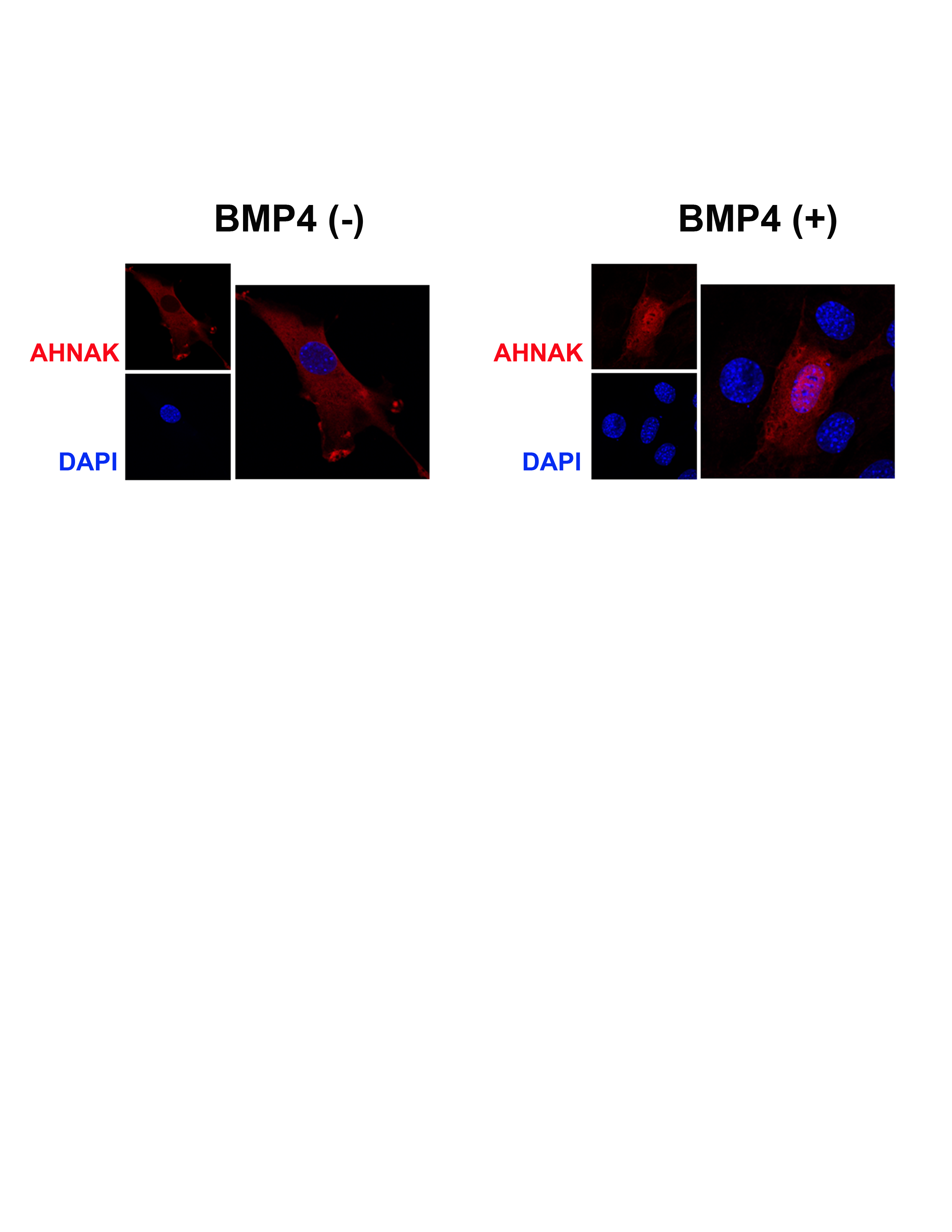

Supplement: S4 Fig — DAPI indicates nuclear staining. (TIF) [file pone.0139720.s004.tif]

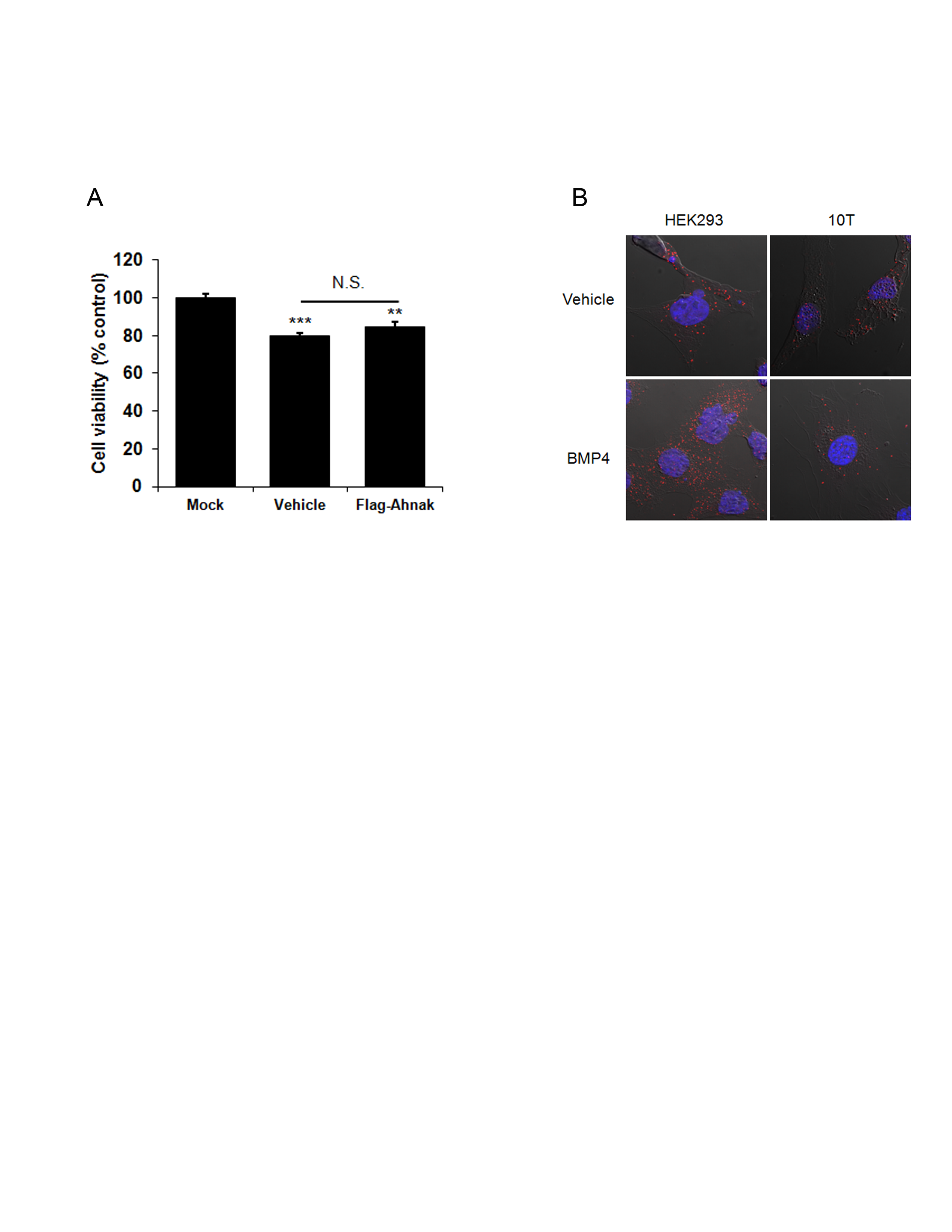

Supplement: S5 Fig — (A) Cell viability following transfection of Flag Ahnak expressed vector in C3H10T cells. (B) The Ahnak-Smad1 complexes were detected as spots in situ Duolink PLA. Nuclear material was stained by DAPI. The data shown are the mean±SEM, *P<0.05, **P<0.01, ***P<0.001 vs Mock. (TIF) [file pone.0139720.s005.tif]

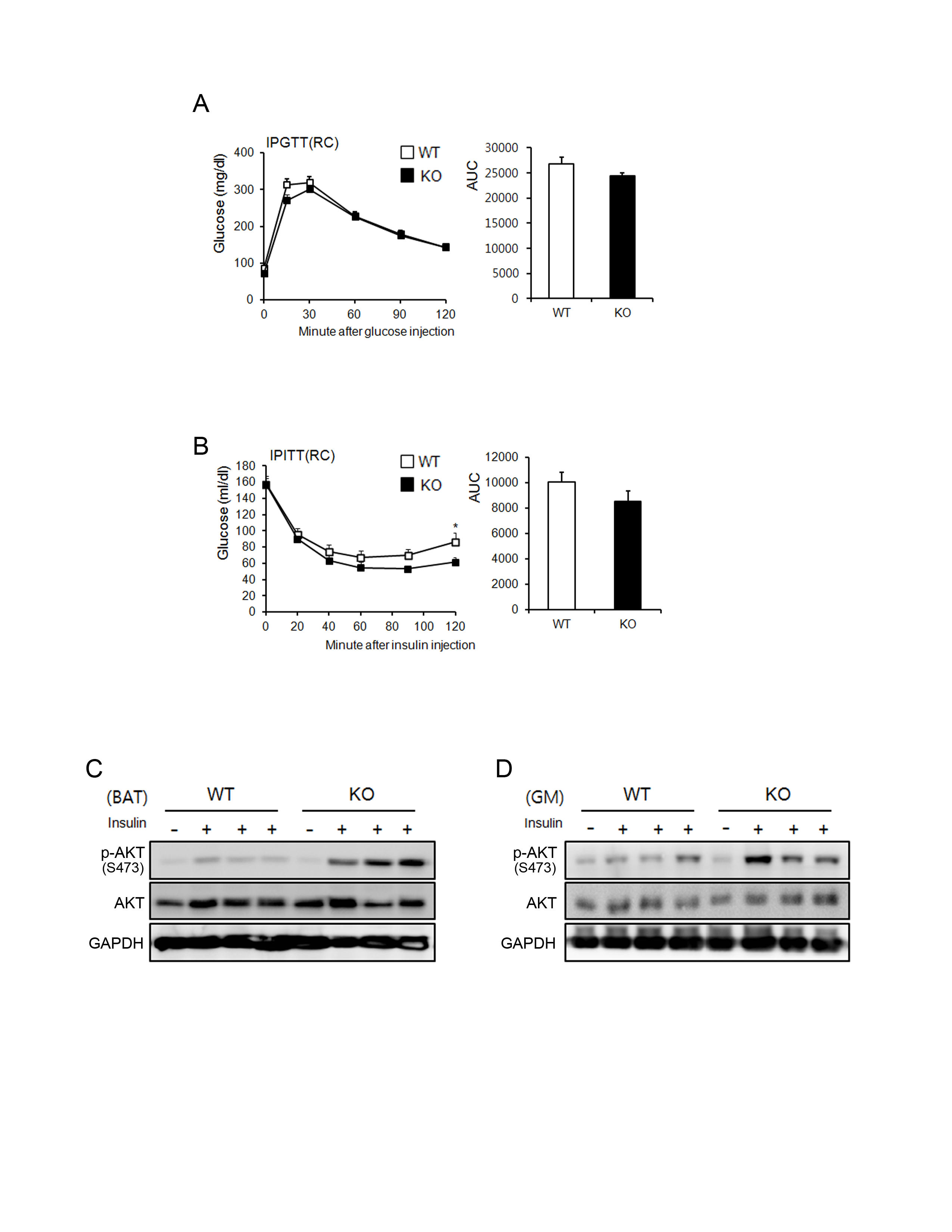

Supplement: S6 Fig — (A) Glucose tolerance after feeding on RC; WT: n = 6, KO: n = 4. (B) Insulin tolerance after of feeding on RC; WT: n = 4, KO: n = 6. The data shown are the mean±SEM, *P<0.05, **P<0.01, ***P<0.001 between WT and KO mice. (C and D) Insulin signaling pathway in BAT (C) and gastrocnemius muscle (GM) (D) after an 8-week HFD. (TIF) [file pone.0139720.s006.tif]

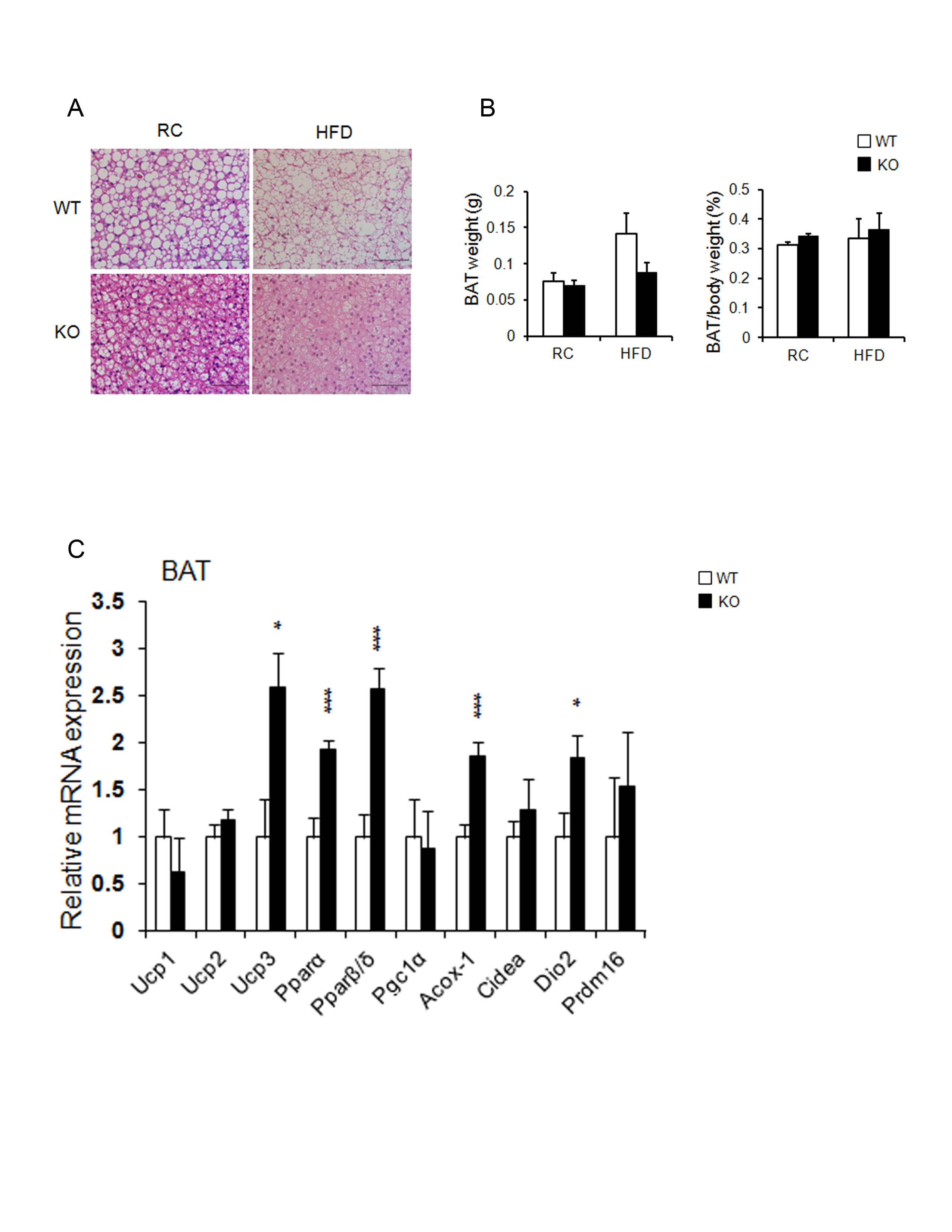

Supplement: S7 Fig — (A) H&E staining of BAT. Scale bar, 200 μm; (B) Quantification of BAT mass (left) and BAT mass normalized by body mass (right); WT: n = 5, KO: n = 4. (C) Relative mRNA expression involved in energy dissipation and brown adipose specific genes in BAT measured by qPCR from mice fed an HFD for 12 weeks (n = 6). Values were normalized to 36B4 expression. The data shown are mean±SEM; *P<0.05, **P<0.01, ***P<0.001. (TIF) [file pone.0139720.s007.tif]

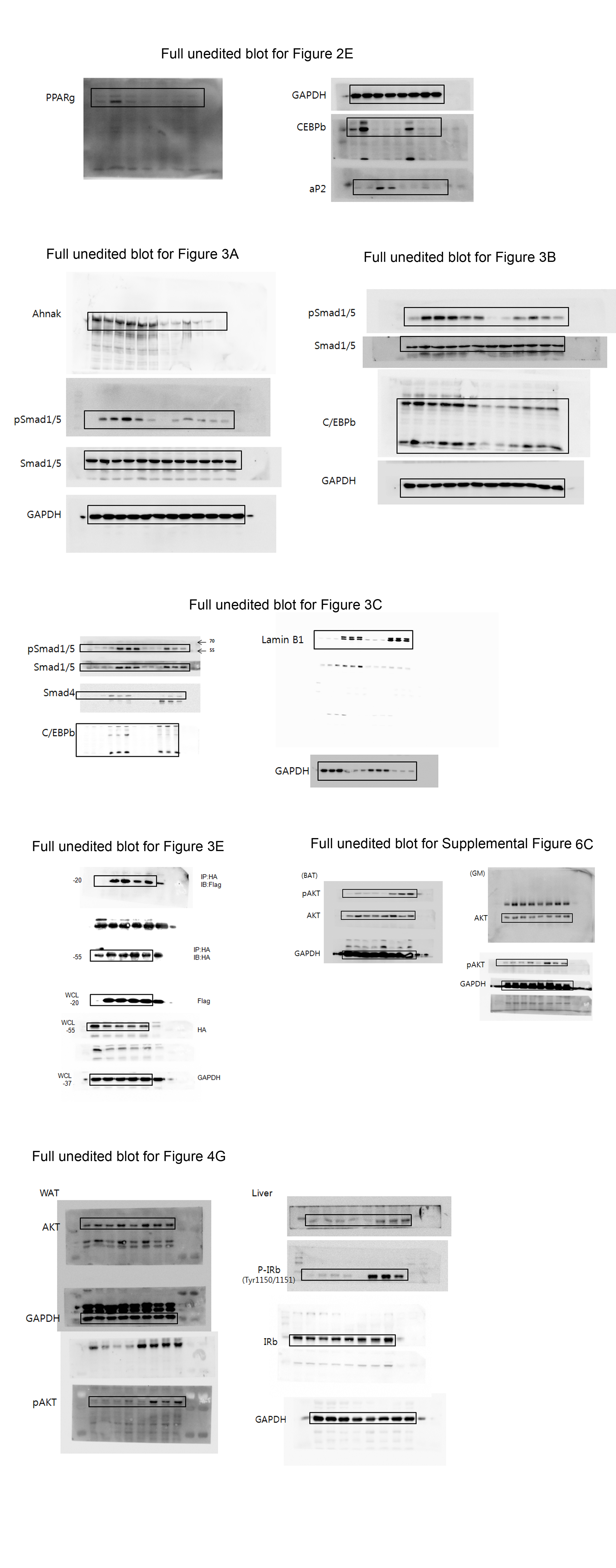

Supplement: S8 Fig — Prestained Protein Marker (GenDEPOT, TX, USA) was used as a size marker. Therefore it is not visible in images obtained by CCD camera. (TIF) [file pone.0139720.s008.tif]
