# Supplementary material for: Obesity Resistance and Enhanced Insulin Sensitivity in Ahnak -/- Mice Fed a High Fat Diet Are Related to Impaired Adipogenesis and Increased Energy Expenditure
Source: PLoS One. 2015 Oct 14;10(10):e0139720. doi: 10.1371/journal.pone.0139720 (PMC4605776; doi:10.1371/journal.pone.0139720)
Supplement: S1 Table — (DOCX) [file pone.0139720.s009.docx]

**Supporting Information**

**Methods**

**In situ proximity ligation assays**

The interactions between Ahnak and Smad1 were determined by performing Duolink^TM^ in situ proximity ligation assays (PLAs; Sigma), according to the manufacturer's instructions. HEK293 and C3H10T1/2 cells were stimulated with BMP4 (25 ng/ml) or not stimulated and then fixed, permeabilized, and incubated with a mouse anti-AHNAK monoclonal antibody in combination with a polyclonal rabbit anti-SMAD antibody, followed by incubation with secondary antibodies conjugated to PLA probes.

**In vivo scanning of abdominal fat**

The entire torso of each mouse was scanned at a resolution of 35 µm by *in vivo* micro computed tomography (micro-CT) (Skyscan 1076, Skyscan Inc., Belgium). Fat tissue, lean tissue (muscle and internal organs), and skin and lumbar vertebrae on acquired two-dimensional cross-section images from each mouse were extracted using a threshold method [1,2]. Three-dimensional models of fat (between the proximal end of L2 (lumbar vertebrae 2) and the distal end of L5) were reconstructed by Mimics software, version 12.0 (Materialise NV, Belgium), and fat volumes were determined.

**Cellular viability assay**

The cell viability was examined by using an EZ-Cytox Cell viability assay kit (Daeil Lab Service, Seoul, Korea). The relative cell viability was determined by measuring the absorbance at 450 nm using a Tecan microplate reader.

**Antibodies**

The primary antibodies used for western blot analysis were specific for: pSMAD1/5, SMAD1, SMAD4, PPARγ, LAMIN B1, GAPDH (Cell Signaling Technologies), and C/EBPβ (Abcam, Cambridge, UK). The antibodies used for immunoprecipitation experiments were AHNAK (Abcam) and SMAD1 (Cell Signaling). A peroxidase-conjugated secondary antibody (Abclon) was also used. Anti-SMAD1/5 (Abcam) and anti-pSmad1/5 (Millipore) antibodies were used for immunostaining. Following secondary antibodies were used: Alexa Fluor 488- or 594-conjugated antibody. DAPI (ImmunoBioScience Co., Washington, USA) was used for nuclear staining.

**Oil red O staining**

Oil red O staining was performed on day 8 following adipogenesis induction. Cells were washed and fixed with 4% paraformaldehyde. The cells were then washed with 60% isopropanol, dried completely, and stained with oil red O (6 parts of 0.5% oil red O powder in isopropanol and 4 parts water) for 10 min and washed with phosphate-buffered saline.

**GEO search for Ahnak expression**

We used the GEO (Gene Expression Omnibus) database to assess Ahnak expression differences in obesity. In this study, we used only GEO dataset (GDS)- samples that were already curated. Those datasets contained normalized expression values for each gene from each sample. Total 30 GDS samples were searched in GEO. The differential expression of the Ahnak gene was identified by t-test method and a P value < 0.05.

**Supplementary Figure legends**

**S1 Fig.** **Physiological characteristics of *Ahnak* KO mice.** (A) Ahnak expression in various tissues from 6-week-old C57BL6 mice (n=4–6). Values were normalized to Gapdh. (B) Comparison of body weights from wild type (*Ahnak*^+/+^) and hetero( *Ahnak*^+/-^) mice (n=3). (C) Body weights of WT and KO mice at various ages. (D) Representative pictures of micro-CT showing abdominal cross-sections, where the white arrows indicate fat. (E) Relative mRNA levels of the indicated genes in WAT of RC-fed mice were measured by qPCR. Values were normalized to *36B4*. The data shown are the mean values±SEM, **P*<0.05, ***P*<0.01, ****P*<0.001.

**S2 Fig. *Ahnak* KO mice are protected from hepatic steatosis-induced by an HFD.** (A) Quantification of liver weights. (B) Quantification of liver triglycerides and FFAs in mice. (C) Representative pictures of H&E and oil red O staining of liver sections. The data shown are means±SEM, n=3–5.**P*<0.05, ***P*<0.01, ****P*<0.001 between WT and KO mice.

**S3 Fig. Loss of Ahnak inhibits adipocyte differentiation *in vitro*.** (A) mRNA expression of Ahnak in the adipocyte fraction (Ad) and stromal vascular fraction (SVF) of eWATs (n=4). (B) mRNA expression of Ahnak during adipogenesis in 3T3-L1 cells. (C) 3T3-L1 cells were transfected with siRNA before differentiation induction and subsequently stained with oil red O.

**S4 Fig. The subcellular localizations of Ahnak in C3H10T cells.** DAPI indicates nuclear staining.

**S5 Fig. Interaction between Ahnak and Smad1.** (A) Cell viability following transfection of Flag Ahnak expressed vector in C3H10T cells. (B) The Ahnak-Smad1 complexes were detected as spots in situ Duolink PLA. Nuclear material was stained by DAPI. The data shown are the mean values ± SEM, **P*<0.05, ***P*<0.01, ****P*<0.001 vs Mock.

**S6 Fig. Insulin signaling**. (A) Glucose tolerance after feeding on RC; WT: *n*=6, KO: *n*=4. (B) Insulin tolerance after of feeding on RC; WT: *n*=4, KO: *n*=6. The data shown are the mean values ± SEM, **P*<0.05, ***P*<0.01, ****P*<0.001 between WT and KO mice. (C and D) Insulin signaling pathway in BAT (C) and gastrocnemius muscle (GM) (D) after an 8-week HFD.

**S7 Fig. Phenotypic characteristics of BAT.** (A) H&E staining of BAT. Scale bar, 200 µm; (B) Quantification of BAT mass (left) and BAT mass normalized by body mass (right); WT: *n*=5, KO: n=4. (C) Relative mRNA expression involved in energy dissipation and brown adipose specific genes in BAT measured by qPCR from mice fed an HFD for 12 weeks (n=6). Values were normalized to *36B4* expression. The data shown are means±SEM; **P*<0.05, ***P*<0.01, ****P*<0.001

**S8 Fig. Full unedited blots used in Figure 1, 3, 4 and Supplement Figures 6.** Prestained Protein Marker (GenDEPOT, TX, USA) was used as a size marker. Therefore it is not visible in images obtained by CCD camera.

**S1 Table. Sequences of primers used for real-time PCR**

| **Gene** | **Sequence (5′–3′)** | **Gene** | **Sequence (5′–3′)** |
| --- | --- | --- | --- |
| Abhd5 | TGGTGTCCCACATCTACATCA | Glut4 | AAAAGTGCCTGAAACCAGAG |
|  | CAGCGTCCATATTCTGTTTCCA |  | TCACCTCCTGCTCTAAAAGG |
| Acox-1 | TCGAAGCCAGCGTTACGAG | LCAD | AACACCATGTATGCTCGGCTC |
|  | ATCTCCGTCTGGGCGTAGG |  | CCGACCAAGATAATCCCCTTGAG |
| Acrp30 | GGCAGGAAAGGAGAACCTGG | LPL | TCTGTACGGCACAGTGG |
|  | AGCCTTGTCCTTCTTGAAGAG |  | CCTCTCGATGACGAAGC |
| Adrb3 | ATTGGCGCTGACTGGCCATT | PGC1α | CCCTGCCATTGTTAAGACC |
|  | CCTCGGCATCTGCCCCTACA |  | TGCTGCTGTTCCTGTTTTC |
| Ahnak | TGTCACTGGCTCACCTGAAG | Plin2 | GACCTTGTGTCCTCCGCTTAT |
|  | CTTGCCTTTAGCGTCTCCAC |  | CAACCGCAATTTGTGGCTC |
| aP2 | TTCGATGAAATCACCGCAGA | PnPla1 | GGTGACCATCTGCCTTCCAG |
|  | AGGGCCCCGCCATCT |  | TGCAGAAGAGACCCAGCAGT |
| CD36 | ATTGGTCAAGCCAGCT | PPARα | TCGGCGAACTATTCGGCTG |
|  | TGTAGGCTCATCCACTAC |  | GCACTTGTGAAAACGGCAGT |
| C/EBPα | GACATCAGCGCCTACATCGA | PPARβ/δ | TTGAGCCCAAGTTCGAGTTTG |
|  | TCGGCTGTGCTGGAAGAG |  | CGGTCTCCACACAGAATGATG |
| C/EBPβ | ATTTCTATGAGAAAAGAGGCGTATGT | PPARγ2 | CCAGAGCATGGTGCCTTCGCT |
|  | AAATGTCTTCACTTTAATGCTCGAA |  | CAGCAACCATTGGGTCAGCTC |
| C/EBPδ | TTCCAACCCCTTCCCTGAT | PRDM16 | CAGCACGGTGAAGCCATTC |
|  | CTGGAGGGTTTGTGTTTTCTGT |  | GCGTGCATCCGCTTGTG |
| Cidea | TGCTCTTCTGTATCGCCCAGT | UCP1 | ACTGCCACACCTCCAGTCATT |
|  | GCCGTGTTAAGGAATCTGCTG |  | CTTTGGCTCACTCAGGATTGG |
| Dgat2 | TCTCAGCCCTCCAAG ACATC | UCP2 | CAGATGTGGTAAAGGTCCGCT |
|  | GCCAGCCAGGTGAAGTAGAG |  | TTCCTCTCGTGCAATGGTCTT |
| Dio2 | CAGTGTGGTGCACGTCTCCAATC | UCP3 | GAGATGGTGACCTACGACATCA |
|  | TGAACCAAAGTTGACCACCAG |  | GCGTTCATGTATCGGGTGTTTA |
| HSL | GGAGCACTACAAACGCAAC | 36B4 | GAGGAATCAGATGAGGATATGGGA |
|  | TCCCGTAGGTCATAGGAGAT |  | AAGCAGGCTGACTTGGTTGC |

**References**

1. Butz F, Ogawa T, Chang TL, Nishimura I (2006) Threedimensional bone-implant integration profiling using microcomputed tomography. Int J Oral Maxillofac Implants 21: 687-695.
2. Rebaudi A, Kolle B, Laib A, Trisi P (2004) Microcomputed tomographic analysis of the peri-implant bone. [Int J Periodontics Restorative Dent](http://www.ncbi.nlm.nih.gov/pubmed/15446401) 24: 316-325.
